# TLR7-dependent eosinophil degranulation links psoriatic skin inflammation to small intestinal inflammatory changes in mice

**DOI:** 10.1038/s12276-024-01225-y

**Published:** 2024-05-01

**Authors:** Hee Joo Kim, Jinsun Jang, Kunhee Na, Eun-Hui Lee, Hyeon-Jung Gu, Yoon Hee Lim, Seul-A Joo, Seung Eun Baek, Joo-Young Roh, Han-Joo Maeng, Yun Hak Kim, Young-Jae Lee, Byung-Chul Oh, YunJae Jung

**Affiliations:** 1 Department of Dermatology, Gachon Gil Medical Center, College of Medicine, Gachon University, Incheon, 21565 Korea; 2 Lee Gil Ya Cancer and Diabetes Institute, Gachon University, Incheon, 21999 Korea; 3 Department of Health Science and Technology, Gachon Advanced Institute for Health Science & Technology, Gachon University, Incheon, 21999 Korea; 4 Department of Microbiology, College of Medicine, Gachon University, Incheon, 21999 Korea; 5 College of Pharmacy, Gachon University, Incheon, 21936 Korea; 6 Department of Anatomy, School of Medicine, Pusan National University, Yangsan, 50612 Korea; 7 Department of Biomedical Informatics, School of Medicine, Pusan National University, Yangsan, 50612 Korea; 8 Department of Biochemistry, College of Medicine, Gachon University, Incheon, 21999 Korea; 9 Department of Physiology, College of Medicine, Gachon University, Incheon, 21999 Korea; 10 Present Address: Department of Dermatology, Ewha Womans University Medical Center, College of Medicine, Ewha Womans University, Seoul, 07804 Korea

**Keywords:** Autoimmunity, Psoriasis

## Abstract

Recent evidence of gut microbiota dysbiosis in the context of psoriasis and the increased cooccurrence of inflammatory bowel disease and psoriasis suggest a close relationship between skin and gut immune responses. Using a mouse model of psoriasis induced by the Toll-like receptor (TLR) 7 ligand imiquimod, we found that psoriatic dermatitis was accompanied by inflammatory changes in the small intestine associated with eosinophil degranulation, which impaired intestinal barrier integrity. Inflammatory responses in the skin and small intestine were increased in mice prone to eosinophil degranulation. Caco-2 human intestinal epithelial cells were treated with media containing eosinophil granule proteins and exhibited signs of inflammation and damage. Imiquimod-induced skin and intestinal changes were attenuated in eosinophil-deficient mice, and this attenuation was counteracted by the transfer of eosinophils. Imiquimod levels and the distribution of eosinophils were positively correlated in the intestine. TLR7-deficient mice did not exhibit intestinal eosinophil degranulation but did exhibit attenuated inflammation in the skin and small intestine following imiquimod administration. These results suggest that TLR7-dependent bidirectional skin-to-gut communication occurs in psoriatic inflammation and that inflammatory changes in the intestine can accelerate psoriasis.

## Skin-Gut link in Psoriasis: Eosinophils accelerate inflammation pathway

Psoriasis is a long-lasting skin disease that involves the body’s immunee system. However, it’s not clear how the immune responses in the skin and gut are connected to psoriasis. In this research, scientists discovered that skin inflammation due to psoriasis in mice causes changes in the small intestine, mainly impacting the immune environment. The research also showed that psoriasis was less severe in mice without eosinophils, a kind of white blood cell. The scientists concluded that there’s a two-way interaction between the skin and the gut in psoriasis, and that the unique immune characteristics of the small intestine are crucial in psoriasis development. This study could possibly lead to new psoriasis treatments.

This summary was initially drafted using artificial intelligence, then revised and fact-checked by the author.

## Introduction

Psoriasis is a chronic immune-mediated skin disease characterized by uncontrolled proliferation of keratinocytes and the infiltration of immune cells^1^. Although the pathogenesis of psoriasis is not fully understood, the immune system is dysregulated by immunogenic stimuli, which activates innate immune cells, resulting in the production of proinflammatory cytokines such as interleukin (IL)-23 and tumor necrosis factor-alpha (TNF-α) and leading to the differentiation of IL-17-secreting T cells^2,3^. These inflammatory changes in the skin activate keratinocytes, amplify the production of inflammatory mediators, and accelerate the immunopathological features of psoriasis^2^. In addition to its pathogenic dermatological features, psoriasis is frequently associated with extracutaneous manifestations, such as psoriatic arthritis and Crohn’s disease^4^. Recent studies have reported imbalances in the gut microbiome and the presence of bacterial DNA derived from the gut microbiome in the sera of patients with psoriasis, suggesting that alterations in the intestinal microenvironment contribute to the pathogenesis of psoriasis^5–7^. However, the mechanisms linking dysregulated immune responses in the skin and gut to psoriasis remain poorly understood.

Although the small and large intestines form a continuous tube, they are distinct microenvironments with different cellular distributions and physiological responses^8^. The small intestine provides a large surface area for nutrient absorption and contains many innate and adaptive immune cells in the lamina propria^9^. These immune cells, including Th17 cells, eosinophils, and plasmacytoid dendritic cells (pDCs), support the integrity of the small intestinal barrier and promote noninflammatory immune tolerance^10–12^. The large intestine is characterized by a high density of IgA^+^ plasma cells and regulatory T (Treg) cells, which protect against the densely populated commensal microbiota in the distal intestinal tract^9,13,14^. The marked immunologic differences in the intestinal tract are important for understanding the complex interactions between the skin and intestine in psoriatic inflammation.

The innate immune system senses pathogenic signals via Toll-like receptors (TLRs)^15^. Topical application of the TLR7 agonist imiquimod (IMQ) to the skin leads to psoriatic skin inflammation in mice, which is associated with many hallmarks of human psoriasis, including increased epidermal proliferation, hyperkeratosis, scaling, and erythema^16^. Using this murine model of psoriasis, we investigated immune responses in the skin and intestine and found that inflammatory changes in the small intestine associated with eosinophil degranulation increased the severity of psoriatic dermatitis. Furthermore, psoriasis-like dermatitis led to eosinophil degranulation and immunological changes in the small intestine in a TLR7-dependent manner. These findings suggest that there is a bidirectional pathogenic interaction between the skin and the gut in psoriasis and that the unique immunological characteristics of the small intestine play a critical role in psoriasis development.

## Materials and Methods

### Human serum

Seventeen patients (7 males and 10 females; mean age: 40.00 ± 14.16 years) diagnosed with psoriasis based on clinical and histologic findings were included in the study. Control serum was collected from 10 healthy volunteers (one male and 9 females: mean age: 38.30 ± 7.60 years). This study was approved by the Ethics Committee of Gachon University Gil Medical Center (GAIRB2017-385) and was conducted according to the Declaration of Helsinki. All the participants provided written informed consent.

### Mice

Six- to eight-week-old female and male BALB/c mice were purchased from Orient Bio (Gyeonggi-do, Korea). ΔdblGATA mice (C.129S1(B6)-*Gata1*^tm6Sho^/J, stock number 005653) and *Ccr3*^-/-^ mice (C.129S4-*Ccr3*^tm1Cge^/J, stock number 005440) were purchased from the Jackson Laboratory (Bar Harbor, ME, USA). *Tlr7*^-/-^ mice (BALB/c.129P2-*Tlr7*^tm1Aki^/Obs) were obtained from Oriental BioService (Kyoto, Japan). Mice expressing Cre under the control of the eosinophil peroxidase (EPX) promoter (*Epx*^Cre/+^; B6.Cg-*Epx*^tm1.1(cre)Jlee^/Jlee)^17^ were obtained from Elizabeth Jacobsen (Mayo Clinic, Phoenix, AZ, USA). *Sirpa*^fl/fl^ mice (*Sirpa*^tm1.1Mato^)^18^ were provided by Takashi Matozaki (Kobe University Graduate School of Medicine, Japan). Eosinophil-specific *Sirpa*^-/-^ mice (*Epx*^Cre/+^*Sirpa*^fl/fl^) were generated by crossing *Sirpa*^fl/fl^ mice with *Epx*^Cre/+^ mice. The ΔdblGATA and *Tlr7*^-/-^ mice had a BALB/c background, and the *Epx*^Cre/+^ and *Sirpa*^fl/fl^ mice had a C57BL/6 background. Eight- to twelve-week-old males and females were used in this study. Littermates were used for experiments with *Epx*^Cre/+^*Sirpa*^fl/fl^ and *Epx*^+/+^*Sirpa*^fl/fl^ mice. ΔdblGATA, *Tlr7*^-/-^, and wild-type mice were not littermates. The mice were maintained at standard temperature and humidity under specific pathogen-free conditions. All procedures involving mice were reviewed and approved by the Center of Animal Care and Use of the Lee Gil Ya Cancer and Diabetes Institute, Gachon University (numbers: LCDI-2020-0113, LCDI-2021-0111, and LCDI-2023-0018).

### IMQ-induced psoriasis-like skin inflammation

At 8–10 weeks of age, the mice received daily topical administrations of 62.5 mg IMQ cream (5%) (Aldara™; 3 M Pharmaceuticals, Maplewood, MN, USA) or vehicle cream (Vaseline, Unilever, Rotterdam, Netherlands) on the shaved back for 5–7 consecutive days.

### Fluorescein isothiocyanate (FITC)-dextran permeability assay

The mice were deprived of water for 16–18 h, after which they were administered a single dose of 44 mg/100 g body weight 4-kDa FITC-dextran (Sigma‒Aldrich, St. Louis, MO, USA; cat# FD4). After 4 h, the FITC-dextran concentration in the serum was measured at 528 nm using a VICTOR X4 multilabel plate reader (PerkinElmer, Waltham, MA, USA).

### Enzyme-linked immunosorbent assay (ELISA)

A Quantikine Mouse CD14 ELISA Kit (R&D Systems, Minneapolis, MN, USA; cat# MC140) and a DuoSet Mouse Calprotectin ELISA Kit (R&D Systems; cat# DY8596-05) were used according to the manufacturer’s instructions. A human zonulin ELISA kit (Elabscience, Houston, TX, USA; cat# E-EL-H5560) was used to measure the serum levels of zonulin according to the manufacturer’s instructions. EPX was measured by sandwich/capture ELISA (anti-EPX capture antibody clone MM25-429.1.13; biotinylated anti-EPX detection antibody clone MM25-82.2.1, provided by Elizabeth Jacobsen), as previously described^19^. The absorbances of soluble CD14, calprotectin, and zonulin were measured at 450 nm, and that of EPX was measured at 630 nm using the VICTOR X4 instrument.

### Cytokine array analysis

The serum profiles of 40 different cytokines were assessed using a Mouse Cytokine Array Kit according to the manufacturer’s instructions (R&D Systems; cat# ARY006). Chemiluminescent signals were measured using an ImageQuant LAS 4000 biomolecular imager (GE Healthcare Bio-Sciences, Uppsala, Sweden). Signal quantification was performed using HLimage++ software (Western Vision Software, Salt Lake City, UT, USA).

### Histology

Skin, small intestine, and large intestine specimens were fixed in 10% neutral-buffered formalin (BBC Biochemical, Mount Vernon, WA, USA) for 24 h and then transferred to 70% ethanol. After dehydration in a series of ethanol gradients and being washed with xylene, the tissues were embedded in paraffin. Multiple 4-μm-thick sections were stained with hematoxylin and eosin. For immunofluorescence staining, fixed small intestine sections were hydrated in alcohol for 30 min and then placed in a 10 mM citric acid solution for 15 min to facilitate antigen recovery. Subsequently, the sections were permeabilized with 0.2% Triton-X-100 at room temperature for 10 min and blocked with 10% normal goat serum (Vector Laboratories, Newark, CA, USA; cat# S-1000-20) for 1 h. The tissue slides were incubated overnight with mucin 2 polyclonal antibody (1:200 dilution, GeneTex, Irvine, CA, USA; cat# GTX100664) or occludin polyclonal antibody (1:100 dilution, Thermo Fisher Scientific, Waltham, MA, USA; cat# 40-4700). The sections were washed with PBS and incubated with Alexa Fluor 488-linked goat anti-rabbit IgG (H&L) secondary antibody (1:250 dilution, Abcam, Cambridge, UK; cat# ab150077) for 1 h. After 10 min of staining with 4’,6-diamidino-2-phenylindole (Thermo Fisher Scientific; cat# D-3571), the slides were mounted using ProLong Gold Antifade (Molecular Probes, Eugene, Oregon, USA). For EPX staining, the intestinal sections were stained with an anti-EPX antibody (MM25-82.2.1; provided by Elizabeth Jacobsen) as previously described^20^. The sections were visualized using a DM6 B microscope equipped with a DFC7000T camera (Leica, Wetzlar, Germany). The fluorescence signals were quantified using i-SOLUTION™ (IMT i‐Solution, Inc., Vancouver, BC, Canada).

### Microbiota analysis

Genomic DNA was extracted from frozen stool samples using the QIAamp Fast DNA Stool Kit (QIAGEN, Hilden, Germany) according to the manufacturer’s instructions. Amplification and sequencing of the 16S rRNA gene were performed as previously described^21^.

### RNA extraction, library construction, and sequencing

RNA was extracted from the skin and small intestines of mice using QIAzol and purified using an RNeasy Mini Kit (QIAGEN). The purified RNA was processed with DNase I (New England Biolabs, Ipswich, MA, USA) to remove genomic DNA. The total RNA concentration was calculated using Quant-IT RiboGreen (Invitrogen, Waltham, MA, USA). To assess the integrity of the total RNA, the samples were examined on a DEa TapeStation RNA screentape (Agilent, Santa Clara, CA, USA) with an RNA integrity threshold ≥ 7. A library was independently prepared with 1 μg of total RNA from each sample using an Illumina TruSeq Stranded mRNA Sample Prep Kit. The libraries were amplified by PCR and sequenced by Macrogen using an Illumina NovaSeq sequencing system to produce 100-bp paired-end reads.

### Sequence annotation and statistical analysis of gene expression

The raw reads were processed to remove low-quality adapter sequences. The processed reads were aligned to the mm10 mouse reference genome using HISAT v2.1.0^22^. StringTie v2.1.3b was used to assemble the aligned reads into transcripts and estimate their abundance. Genes with read counts of zero in at least one sample were excluded. To facilitate log2 transformation, 1 was added to each read count of the filtered genes. The filtered data were log2-transformed and normalized to the relative log expression. Statistically significant differentially expressed genes were determined by the nbinomWald test using DESeq2 and fold change. The null hypothesis was that there was no difference among the groups.

### Gene set enrichment analysis (GSEA)

GSEA was performed using GSEA v4.3.2, provided by the Broad Institute (Cambridge, MA, USA) as previously described^23^. Enrichment analysis was performed using hallmark gene sets from the MsigDB database. To determine the enrichment of the ontology gene sets (C5.all.v2022.1), mouse gene symbols were remapped to human orthologs. Leading-edge analysis was performed to determine overlapping gene sets. Selected gene sets with *P* < 0.05 and a false discovery rate < 0.25 are shown.

### Preparation of small intestine cell suspensions

After removing the fat and Peyer’s patches, the small intestine was opened longitudinally, flushed with PBS, and cut into segments. The segments were incubated with FACS buffer containing PBS, 5% fetal bovine serum (FBS; Gibco, Waltham, MA, USA), 20 mM hydroxyethyl piperazine ethane sulfonic acid (HEPES; Gibco), 1 mM sodium pyruvate (Gibco), and 10 mM ethylene diamine tetraacetic acid (LPS Solution, Daejeon, Korea) for 30 min at 37 °C to remove epithelial cells and then washed extensively with PBS. The FACS buffer-treated intestinal segments were thoroughly minced and incubated with 0.2% collagenase D (Roche, Mannheim, Germany) and 10 μg/ml DNase I (Roche) in RPMI 1640 (Sigma‒Aldrich) supplemented with 5% FBS with continuous stirring at 37 °C for 30 min. After being washed, the cells were subjected to density-gradient centrifugation in 40%/75% Percoll (Cytiva, Marlborough, MA, USA). The cells harvested from the interface were washed and used for assays.

### Flow cytometry and cell sorting

To characterize cell surface markers, the isolated cells were resuspended in FACS buffer and incubated with anti-mouse CD16/CD32 (BioLegend, San Diego, CA, USA; cat# 101330) for 15 min at 4 °C. The cells were then stained for 30 min at 4 °C with monoclonal antibodies (mAbs) against Ly-6G phycoerythrin (PE; cat# 551461), CD11c PE (cat# 557401), SiglecF PE (cat# 552126), SiglecF Brilliant Violet 650 (cat# 740557), Ly-6C FITC (cat# 553104) (all from BD Biosciences, San Diego, CA, USA), IA-IE Alexa700 (cat# 56-5321-82), SIRPα PE (cat# 12-1721-80) (eBioscience; San Diego, CA), CCR3 FITC (cat# FAB729F) (R&D Systems), Annexin V allophycocyanin (APC; cat# 640920), 7-aminoactinomycin D (7-AAD; cat# 420403), Zombie Aqua Brilliant Violet 510 (cat# 423102), CD45 PerCP-cy5.5 (cat# 103132), CD4 PE (cat# 100512), CD8a FITC (cat# 100705), CD317 FITC (cat# 127008), B220 APC (cat# 103212), TCR β chain FITC (cat# 109206), TCR γ/δ chain PE (cat# 118108), CD64 APC (cat# 139305), CD11b Brilliant Violet 421 (cat# 101235), and CD63 APC (cat# 143906) (BioLegend). For bromodeoxyuridine (BrdU) labeling experiments, the cells were fixed and stained using an APC BrdU Flow Kit (BD Biosciences; cat# 552598) according to the manufacturer’s instructions. Each sample was analyzed with a FACSymphony or LSRII flow cytometer (BD Biosciences). Small intestinal eosinophils (CD45^+^IA-IE^-^CCR3^+^SiglecF^+^) were sorted using a FACSAria II (BD Biosciences). The data were processed using FlowJo software (Tree Star, Ashland, OR, USA).

### BrdU administration and calculation of the eosinophil half-life

The mice were initially injected with 2 mg of BrdU and subsequently received 0.8 mg/ml BrdU (Sigma‒Aldrich, cat# B5002) in their drinking water for 7 days. The BrdU in the water was replaced every 3 days. The eosinophil half-life (*t*_1/2_) in the small intestine was calculated as *t*_1/2_ = *t*/log_0.5_(*N*_*t*_/*N*_*0*_), where *t* is the time of BrdU administration (7 days), *N*_*t*_ is the percentage of BrdU^-^ eosinophils after 7 days, and *N*_*0*_ is the percentage of BrdU^-^ eosinophils on Day 0 (100%), as previously described^24^.

### Quantitative PCR

cDNA was synthesized from the extracted RNA using an iScript cDNA synthesis kit (Bio-Rad Laboratories, Hercules, CA, USA). Quantitative PCR was performed using the iQ SYBR Green Supermix (Bio-Rad Laboratories) on a CFX Connect real-time PCR detection system (Bio-Rad Laboratories). Relative gene expression was determined using the 2^−ΔΔCt^ method^25^. The primers used are listed in Supplementary Table 1.

### Cell culture

The AML 14.3D10 human eosinophil cell line (kindly provided by Il Yup Chung, Hanyang University, Korea) and Caco-2 human intestinal epithelial cells (Korea Cell Line Bank, Seoul, Korea) were maintained at a density of 10^6^ cells/ml in RPMI 1640 medium (Sigma‒Aldrich) supplemented with 10% FBS, 10 mM HEPES, 100 U/ml penicillin‒streptomycin (Gibco), 50 μg/ml gentamycin (Gibco), 1 mM sodium pyruvate, 50 μM 2-mercaptoethanol (Gibco), and 1 mM nonessential amino acids (Gibco) in an atmosphere with 5% CO_2_ at 37 °C. The culture supernatant was collected from 100% confluent AML14.3D10 cells cultured with or without further stimulation with 10 μg/ml or 100 μg/ml R837 (InvivoGen, San Diego, CA, USA) for 24 h. The supernatant was used as conditioned media (CM). Caco-2 cells were stimulated with CM from AML14.3D10 cells for 24 h.

### Cell viability assay

The viability of Caco-2 cells was determined using a water-soluble tetrazolium salt assay kit according to the manufacturer’s instructions (EZ-Cytox Cell Viability Assay Kit; DoGEN, Seoul, Korea; cat# EZ-1000). The absorbance was measured at 450 nm by the VICTOR3 instrument.

### Analysis of transepithelial electrical resistance (TEER)

Caco-2 cells were seeded on tissue-culture polycarbonate membrane filters (pore size, 0.4 μm) in 24-well Transwell plates (SPL, Gyeonggi, Korea) at a density of 1.5×10^5^ cells/cm^2^. The cells were maintained for 14 days after seeding. Prior to TEER measurements, the standard medium was replaced with CM collected from AML14.3D10 cells. TEER was measured using a Millicell ERS meter (Millipore, Bedford, MA, USA) and calculated as the percentage change in Ω cm^2^ compared with that in the control group.

### Culture of bone marrow-derived eosinophils (BMEOs)

Bone marrow cells were collected and filtered through a 100-μm cell strainer (Corning, Glendale, AZ, USA). Red blood cells were lysed using ammonium chloride potassium lysis buffer. The isolated bone marrow cells were cultured for 14 days. The culture medium was supplemented with 100 ng/ml FMS-like tyrosine kinase 3 ligand and stem cell factor (PeproTech, Rocky Hill, NJ, USA) for the first 4 days, followed by 10 ng/ml IL-5 (PeproTech) for the next 8 days^26^. For the adoptive transfer assay, 1×10^7^ cultured BMEOs were injected intravenously into ΔdblGATA mice.

### Measurement of blood perfusion changes in the skin

Blood perfusion in the skin was examined using a laser Doppler perfusion imaging analyzer (Moor Instruments, Devon, UK) prior to the application of the vehicle cream and IMQ on each day of the experiment. The results are shown as arbitrary perfusion units. The analysis was performed on images from one mouse per group. The regions of interest were selected according to the area of the dorsal skin.

### Liquid chromatography-tandem mass spectrometry (LC‒MS/MS) analysis

An Agilent Technologies HPLC 1200 system coupled to an AB SCIEX Triple Quad 5500 mass spectrometer equipped with a turbo ion spray interface in positive ionization mode (Applied Biosystems SCIEX, Concord, ON, Canada) was used for LC‒MS/MS. Chromatography was achieved in isocratic mode using a mobile phase consisting of 0.1% formic acid in water and acetonitrile (30:70, v/v) at a flow rate of 0.2 ml/min on a SynergiTM 4 μm polar-RP 80 A column (75 mm × 2.0 mm, 2.6 μm; Phenomenex, Torrance, CA, USA) maintained at 30 °C. The autosampler was maintained at 4 °C, and the injection volume was 10 μl for mouse plasma and tissue samples. The selected reaction monitoring transitions were *m/z* 241.0 → 168.0 for IMQ and 180.2 → 162.2 for the internal standard (phenacetin). The mass data were processed using Analyst software version 1.5.2 (Applied Biosystems-SCIEX). The limit of quantitation of IMQ was 1 ng/ml.

### Graphical illustrations

Schematics of the experimental workflows were created using a licensed version of Biorender.com.

### Statistical analysis

All the experiments were performed in duplicate, except for the microbiota and RNA sequencing experiments. Two-group comparisons were performed using a two-tailed unpaired Student’s *t* test or the Mann–Whitney U test. Differences among groups were examined for statistical significance using one-way analysis of variance (ANOVA) with Tukey’s or Bonferroni post hoc correction or the Kruskal–Wallis test with Dunn’s multiple comparison test. Multiple comparison tests were performed using two-way ANOVA with Bonferroni post hoc correction. *P* < 0.05 was considered to indicate statistical significance. Statistical tests were performed based on the normality of the data distribution.

## Results

### Psoriatic skin inflammation in mice induces systemic changes and primarily affects the immune environment of the small intestine

**Fig. 1:**
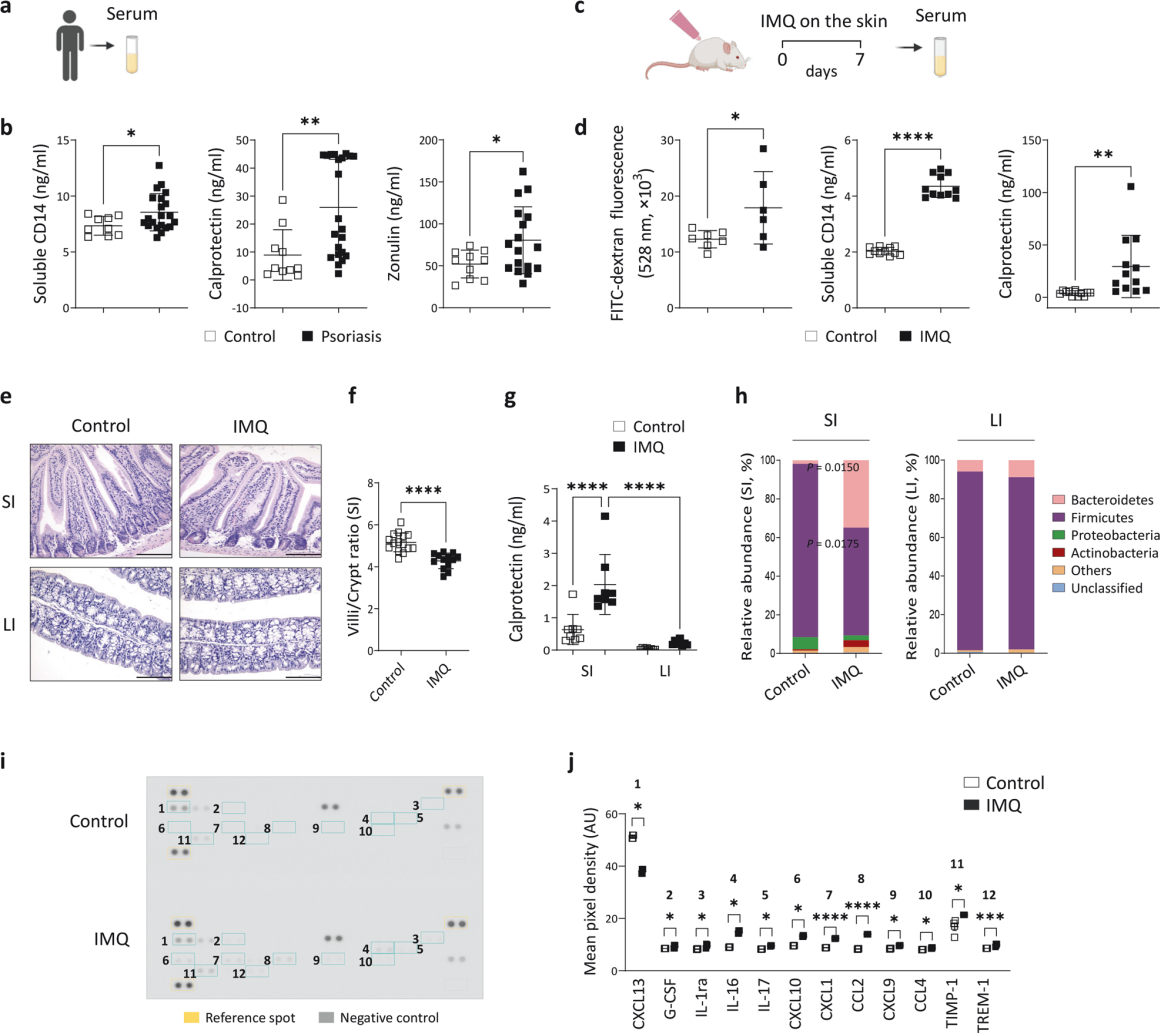
Psoriatic skin inflammation induces changes in the intestinal microenvironment. **a**, **b** Serum concentrations of soluble CD14, calprotectin, and zonulin in healthy individuals and patients with psoriasis. **c** Schematic of imiquimod (IMQ)-induced psoriatic inflammation. **d** Serum fluorescein isothiocyanate (FITC)-dextran fluorescence and soluble CD14 and calprotectin concentrations in mice. **e** Images of hematoxylin and eosin-stained tissues from the small intestine (SI) and large intestine (LI). Scale bars, 124.5 μm. **f** Ratio of villus length to crypt length. Three to five villi and crypts were analyzed per section. **g** Calprotectin concentrations in stool. **h** Sequencing of the stool microbiota of the SI and LI of mice treated with IMQ (*n* = 3) or vehicle cream (*n* = 4). Image of the cytokine array membrane (**i**) and the quantified density (**j**). The data are presented as the means ± SDs. *P < 0.05, **P < 0.01, ***P < 0.001, and ****P < 0.0001 according to unpaired *t* tests (**b**, **d**, **f**, **h**, and CCL2 and CXCL9 in **j**), Mann–Whitney tests (calprotectin in **b** and **j**), or one-way ANOVA with Bonferroni’s multiple comparisons (**g**).

Compared with healthy individuals, patients with psoriasis had increased serum levels of soluble CD14 (sCD14) and calprotectin, which are markers of inflammation^27,28^ (Fig. 1a, b). Upregulated expression of zonulin, which is a biomarker of intestinal barrier integrity^29^, was also observed in the sera of these patients (Fig. 1b). Given that the application of IMQ to mice results in psoriasis-like symptoms^30^, we assessed whether topical IMQ could affect intestinal barrier integrity. As shown in Fig. 1c and d, mice treated with IMQ and administered FITC-dextran by oral gavage exhibited elevated serum levels of FITC-dextran. Additionally, the serum levels of sCD14 and calprotectin were increased in IMQ-treated mice. These findings suggest an increase in intestinal permeability in mice with psoriatic skin inflammation.

Although the histological features of the large intestine were comparable between control and IMQ-treated mice, the small intestines of mice with psoriatic dermatitis exhibited abnormal villous architecture, which was accompanied by a significant decrease in the villus length-to-crypt length ratio (Fig. 1e, f). Consistently, the increased levels of stool calprotectin and alterations in microbial composition were more pronounced in the small intestines (Fig. 1g, h). The significantly increased levels of various inflammatory mediators in the sera of IMQ-treated mice (Fig. 1i, j; Supplementary Fig. 1a) indicated a systemic effect of psoriatic inflammation. To determine whether psoriatic inflammation in mice leads to generalized extracutaneous changes rather than those specific to the small intestine, we assessed the histological characteristics and expression of inflammatory mediators in the kidney and liver. As shown in Supplementary Fig. 1b, the histological structures of these organs were similar between the control and IMQ-treated groups. In addition, we found that the levels of calprotectin and IL-1β per milligram of tissue were increased in the lysates of the small intestine (calprotectin, *P* = 0.0590; IL-1β, *P* = 0.0470) but not in kidney or liver lysates (Supplementary Fig. 1c).

### Psoriatic skin inflammation induces distinct transcriptome changes in the small intestine and skin

**Fig. 2:**
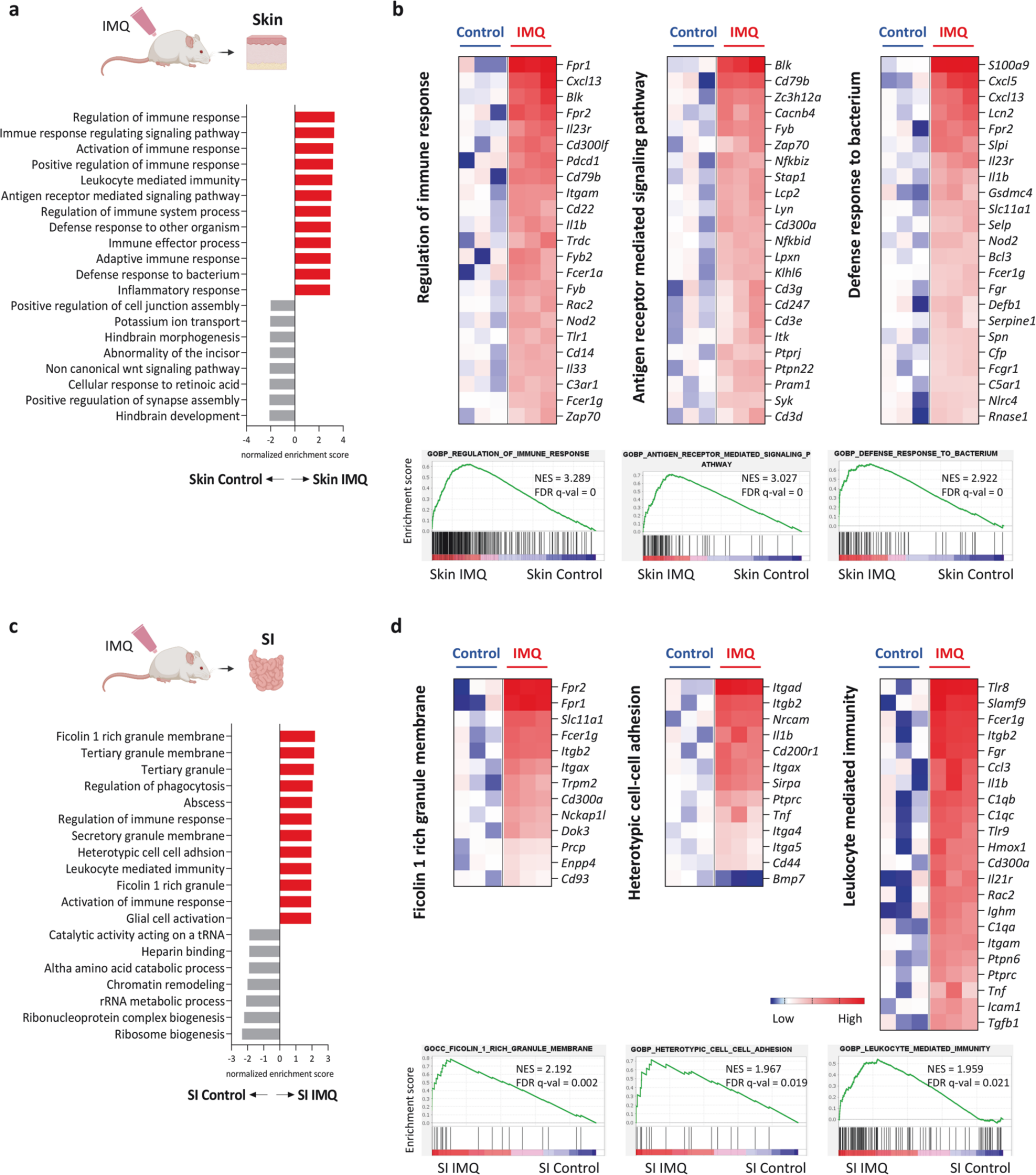
The skin and small intestines of mice with psoriatic inflammation exhibit differential transcriptomic changes. Gene ontology terms enriched in the skin (**a**) and small intestine (SI; **c**) of imiquimod (IMQ)-treated mice compared with vehicle-treated control mice were determined by gene set enrichment analysis. Selected gene sets and enrichment plots of genes that were differentially expressed in the skin (**b**) and SI (**d**) of vehicle-treated and IMQ-treated mice (adjusted *P* < 0.05). The heatmaps show the log_2_-fold change relative to the geometric mean fragments per kilobase of transcript per million mapped reads + 0.01.

To better understand the changes in the small intestines of mice with psoriatic inflammation, we compared the transcriptomes of the skin and the small intestine. GSEA identified gene ontology (GO) terms that were enriched in the skin and small intestines of mice with psoriatic dermatitis compared to those of control mice (Fig. 2a, c). Most of the significant GO terms in the lesional skin were related to inflammatory responses, and genes such as *Cxcl5*, *Il23r*, *Cd3*, and *Zap70*, which are implicated in neutrophil recruitment and Th17 differentiation, were upregulated (Fig. 2b). The significant GO terms in the small intestine were also enriched in immune functions, such as the regulation of phagocytosis and leukocyte-mediated immunity (Fig. 2c). *Tlr8*, *Tlr9*, *C1qb*, *C1qc*, and *Tnf* were among the most highly upregulated IMQ-induced genes related to leukocyte-mediated immunity in the small intestine, indicating the activation of innate inflammation mediated by endosomal TLR-sensing of nucleic acids (Fig. 2d). The small intestines of IMQ-treated mice also exhibited upregulation of several GO terms related to granules or granule membranes (Fig. 2c), suggesting an increase in the release of intracellular granular contents. These findings suggest that psoriatic skin inflammation leads to disturbances in the homeostatic microenvironment of the small intestine, which are distinct from the inflammatory changes in the skin.

### Psoriatic skin inflammation reduces the molecules supporting barrier integrity and decreases the eosinophil count in the small intestine

**Fig. 3:**
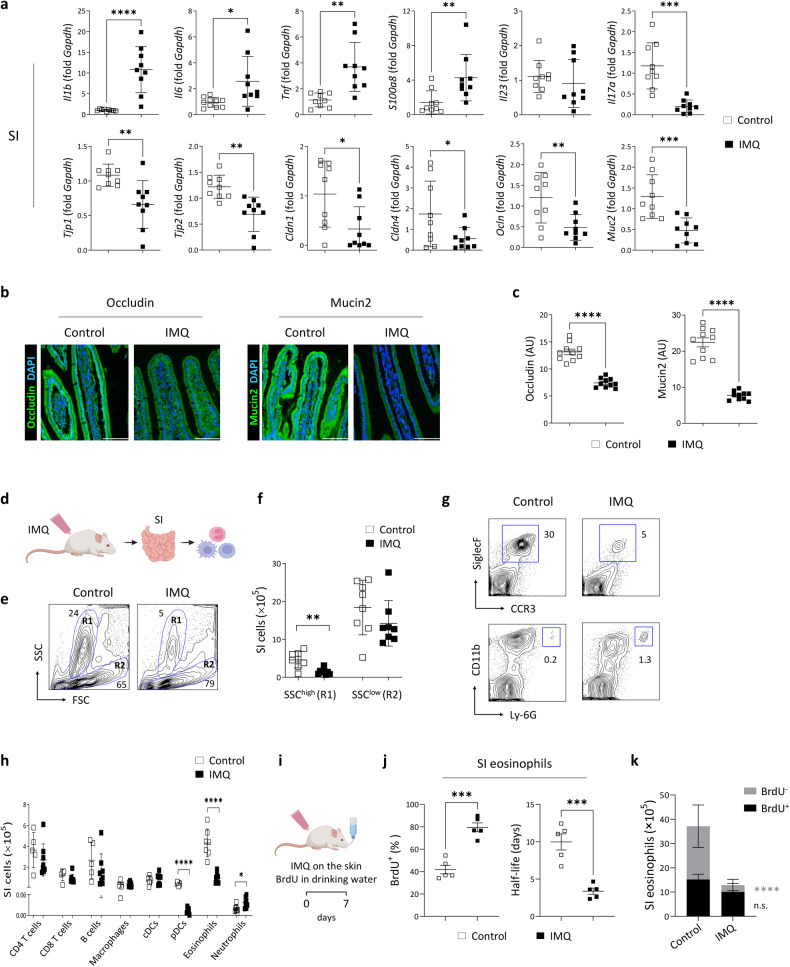
Psoriatic skin inflammation induces inflammatory changes and reduces the number of eosinophils in the small intestine. **a** Quantitative PCR analysis of the small intestine (SI). Occludin and mucin 2 (green) immunofluorescence staining (**b**) and quantification (**c**) in the SI. Nuclei were stained with 4’,6-diamidino-2-phenylindole (DAPI; blue). The fluorescence intensity of each section was analyzed. Scale bars, 62.3 μm. **d** Schematic of the cell isolation procedure. **e** The forward scatter (FSC) and side scatter (SSC) populations of cells in the SI. SSC^high^ and SSC^low^ cells are indicated as R1 and R2, respectively. **f** Absolute number of cells. **g** Representative flow cytometry plots of eosinophils (upper row) and neutrophils (lower row) in the SI. **h** Absolute numbers of immune cells in the SI. cDCs, conventional dendritic cells; pDCs, plasmacytoid DCs. **i** Schematic of bromodeoxyuridine (BrdU) treatment. **j** Percentage of BrdU^+^ eosinophils in the SI (left) and estimated half-life of small intestinal eosinophils (right). **k** The absolute numbers of BrdU^+^ and BrdU^-^ eosinophils in the SI. The data are presented as the mean ± SD. *P < 0.05, **P < 0.01, ***P < 0.001, and ****P < 0.0001 according to unpaired *t* tests (**a**, **c**, **f**, **h**, and **j**), Mann–Whitney tes*t*s (*S100a8* and *Cldn1* in **a**), or two-way ANOVA with Bonferroni’s multiple comparisons (**k**).

IMQ treatment did not increase the expression of *Il23* or *Il17a* in the small intestine, although it increased the expression of several inflammatory cytokines implicated in early innate immune responses (Fig. 3a). In addition, the small intestines of mice with psoriatic inflammation exhibited reduced expression of genes encoding epithelial tight junction molecules (Fig. 3a). Immunofluorescence staining confirmed the reduced protein expression of occludin and mucin2 in the small intestine of mice with psoriatic inflammation (Fig. 3b, c).

Flow cytometric analysis of small intestinal immune cells revealed that IMQ treatment reduced the frequency and total number of cells with high side scatter (SSC^high^) corresponding to granulocytes (Fig. 3d–f). Notably, the frequency and number of eosinophils were dramatically reduced in the small intestines of IMQ-treated mice, whereas those of neutrophils were increased (Fig. 3g, h**;** Supplementary Fig. 2). The ingestion of topically applied IMQ during the development of psoriatic inflammation can lead to intestinal changes^31^. To address this, we analyzed the pathological changes in the skin and small intestines of IMQ-treated mice wearing Elizabethan collars to prevent ingestion. As shown in Supplementary Fig. 3, these mice exhibited genetic expression patterns similar to those of mice not wearing Elizabethan collars and exhibited decreases in the frequency and number of small intestinal eosinophils (Supplementary Fig. 3g, h). These findings indicated that the results were not due to the unintended ingestion of IMQ.

Since eosinophils in the small intestine are terminally differentiated, the reduction in eosinophil numbers may be due to reduced eosinophil migration or defective eosinophil survival^24^. To distinguish between these possibilities, we continuously administered the nucleoside analog BrdU to mice during IMQ treatment and quantified the number of BrdU^+^ and BrdU^-^ eosinophils in the small intestine. As shown in Fig. 3i and j, approximately 42% and 80% of eosinophils were BrdU^+^ in control mice and IMQ-treated mice, respectively, corresponding to an eosinophil half-life of 10.0 days in control mice and 3.4 days in mice with psoriatic dermatitis. Despite an increase in the percentage of BrdU^+^ eosinophils in IMQ-treated mice, the absolute number of BrdU^+^ eosinophils was similar between IMQ-treated and control mice (Fig. 3k). These findings indicate that the survival of small intestinal eosinophils is reduced in mice with psoriatic dermatitis. However, we did not observe a decrease in small intestinal eosinophils or inflammatory changes in the small intestine following intraperitoneal administration of IMQ (Supplementary Fig. 4).

### Degranulation of small intestinal eosinophils induced by psoriatic skin inflammation leads to intestinal epithelial damage

**Fig. 4:**
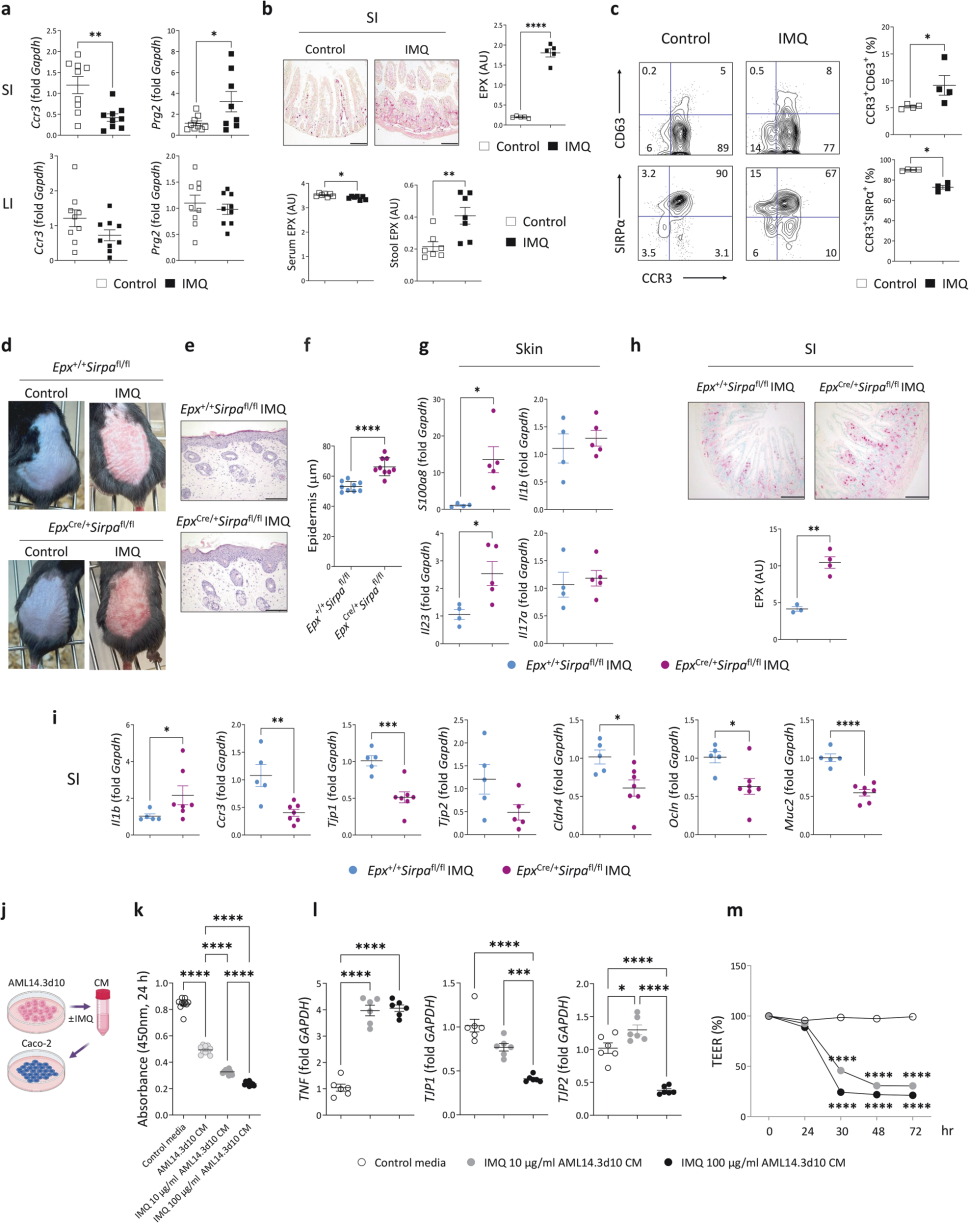
Psoriatic skin inflammation induces the degranulation of small intestinal eosinophils. **a** Quantitative PCR analysis. SI, small intestine; LI, large intestine. **b** Immunohistochemical staining for eosinophil peroxidase (EPX) (upper row) in the SI and EPX levels in the serum and stool (lower row). Scale bars, 134 μm. **c** Representative flow cytometry plots (left) and percentages (right) of CCR3^+^CD63^+^ and CCR3^+^SIRPα^+^ eosinophils in the SI. Singlet SSC^high^CD45^+^CD11b^+^ cells were gated. **d** Photographs of skin treated with vehicle cream (control) or imiquimod (IMQ). **e** Hematoxylin and eosin-stained skin. Scale bars, 124.5 μm. **f** Epidermal thickness. Three distinct regions of each section were analyzed. **g** Quantitative PCR analysis of the skin. **h** EPX immunohistochemical staining and quantification in the SI. The intensity of each section was analyzed. Scale bars, 124.5 μm. **i** Quantitative PCR analysis of the SI. **j** Schematic of Caco-2 cell treatment with conditioned media (CM) collected from AML 14.3D10 cells. **k** Viability of Caco-2 cells. **l** Quantitative PCR analysis of Caco-2 cells. **m** Transepithelial electrical resistance (TEER) of polarized Caco-2 cells. ****P < 0.0001 vs. control media. The data are presented as the mean ± SD. *P < 0.05, **P < 0.01, ***P < 0.001, and ****P < 0.0001 according to the unpaired *t* test (**a**, **b**, **f**, **g**, **h**, and **i**), Mann–Whitney test (**c** and *Il1b* in **i**), one-way ANOVA with Tukey’s multiple comparisons (**k** and **l**), or two-way ANOVA with Bonferroni’s multiple comparisons (**m**).

In eosinophilic inflammatory diseases, eosinophils undergo cytolytic death and induce tissue damage through the release of toxic cytoplasmic granules^32^. We hypothesized that psoriatic skin inflammation disrupted the small intestinal barrier by inducing the release of cytoplasmic granules from cytolytic eosinophils. Topical IMQ administration reduced the expression of *Ccr3*, which is a gene encoding an eosinophil-expressed chemokine receptor, and increased the expression of *Prg2*, which is a gene encoding an eosinophil granule-derived major basic protein, in the small intestine but not in the large intestine (Fig. 4a). Consistent with these findings, mice with psoriatic inflammation exhibited increased levels of eosinophil granule-derived proteins in the intestines, as evidenced by increased EPX expression in the small intestine and stool (Fig. 4b). Eosinophil exocytosis is associated with an increase in the tetraspanin CD63^33^. Conversely, the inhibitory receptor signal regulatory protein alpha (SIRPα) plays a role in suppressing the degranulation of small intestinal eosinophils^34^. Interestingly, we observed that the eosinophils isolated from the small intestines of mice with psoriatic inflammation exhibited increased levels of CD63 and reduced levels of SIRPα compared with those isolated from control mice (Fig. 4c). To determine the causal role of small intestinal eosinophil degranulation in psoriasis, we generated eosinophil-specific SIRPα conditional knockout mice (*Epx*^Cre/+^*Sirpa*^fl/fl^) (Supplementary Fig. 5a and b) and treated them with IMQ. After 5 days of treatment, compared to *Epx*^+/+^*Sirpa*^fl/fl^ mice, *Epx*^Cre/+^*Sirpa*^fl/fl^ mice exhibited exacerbated psoriatic dermatitis characterized by increases in skin thickness and the expression of *S100a8* and *Il23* (Fig. 4d–g). Under steady-state conditions, *Epx*^Cre/+^*Sirpa*^fl/fl^ mice maintained an immune cell composition similar to that of the *Epx*^+/+^*Sirpa*^fl/fl^ mice (Supplementary Fig. 5c). Furthermore, no significant increase in inflammatory gene expression or decrease in the expression of genes linked to barrier function was observed (Supplementary Fig. 5d). However, in response to IMQ treatment, the small intestines of *Epx*^Cre/+^*Sirpa*^fl/fl^ mice exhibited increased EPX and *Il1b* expression and decreased expression of *Ccr3* and genes related to epithelial junctions, including *Tjp1*, *Tjp2*, *Cldn4*, *Ocln*, and *Muc2* (Fig. 4h, i). These findings reinforce the pathological significance of eosinophil degranulation and inflammatory alterations in both the skin and small intestine.

To verify that eosinophil granules induced damage to intestinal epithelial cells, we cultured Caco-2 colon epithelial cells with the culture supernatants of untreated or IMQ-stimulated AML14.3D10 cells (Fig. 4j). AML14.3D10 cells secreted eosinophil-specific granule proteins without differentiation signals, as previously reported (Supplementary Fig. 6)^35^. These supernatants decreased the viability of Caco-2 cells, as indicated by a decrease tetrazolium salt cleavage, reflecting a reduction in mitochondrial dehydrogenase activity (Fig. 4k). Furthermore, compared with cells cultured in control media, Caco-2 cells cultured in IMQ-stimulated AML14.3d10 supernatant exhibited increased expression of inflammatory mediators and decreased expression of tight junction molecules (Fig. 4l). Accordingly, treatment of Caco-2 cells with IMQ-stimulated AML14.3d10 supernatants resulted in a decrease in TEER, indicating increased cell layer permeability (Fig. 4m).

### The absence of eosinophils attenuates skin inflammation and prevents the increase in intestinal permeability

**Fig. 5:**
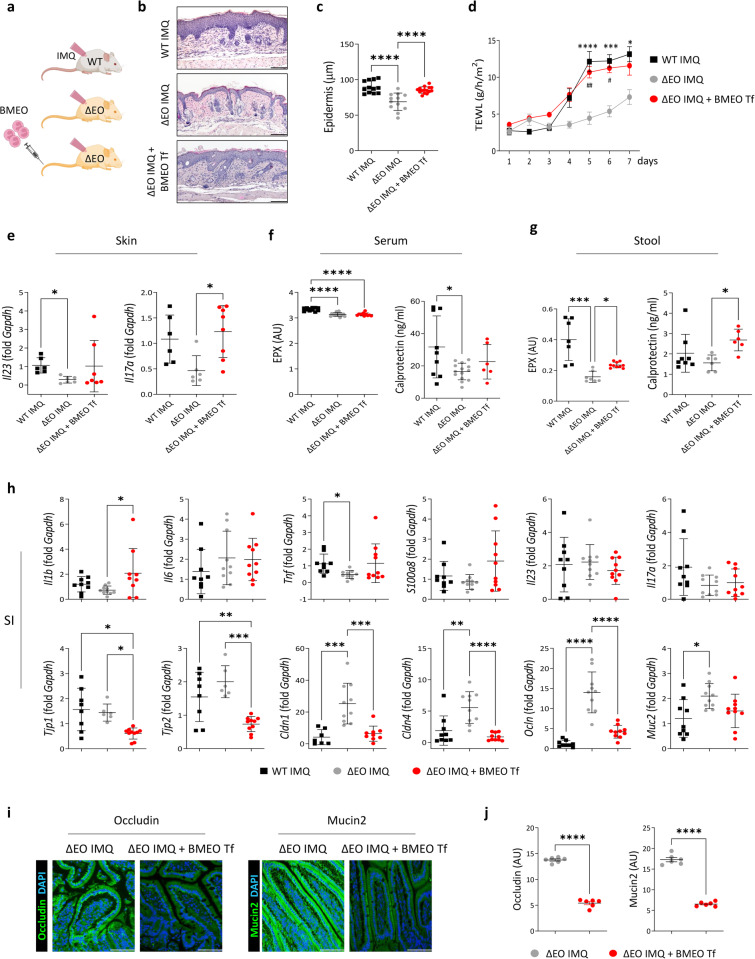
Psoriatic skin inflammation and the increase in intestinal permeability are attenuated in eosinophil-deficient mice. **a** Schematic of the adoptive transfer (Tf) of bone marrow-derived eosinophils (BMEOs). WT, wild-type; ΔEO, ΔdblGATA. (**b**) Hematoxylin and eosin-stained skin. Scale bars, 124.5 μm. **c** Epidermal thickness. Three distinct regions of each section were analyzed. **d** Transepidermal water loss (TEWL) (*n* = 9 or 10/group). *P < 0.05, ***P < 0.001, and ****P < 0.0001, WT IMQ vs. ΔEO IMQ; ^#^*P* < 0.05, ^##^*P* < 0.01, ΔEO IMQ + BMEO Tf vs. ΔEO IMQ. **e** Quantitative PCR analysis of the skin. **f** Serum concentrations of eosinophil peroxidase (EPX) and calprotectin. **g** Stool concentrations of EPX and calprotectin. **h** Quantitative PCR analysis of the small intestine (SI). Occludin and mucin 2 (green) immunofluorescence staining (**i**) and quantification (**j**) in the SI. Nuclei were stained with 4’,6-diamidino-2-phenylindole (DAPI; blue). The fluorescence intensity of each section was analyzed. Scale bars, 62.3 μm. The data are presented as the means ± SDs. *P < 0.05, **P < 0.01, ***P < 0.001, and ****P < 0.0001; one-way ANOVA with Tukey’s multiple comparisons (**c**, **e**, **f**, and **h**); Kruskal–Wallis test with Dunn’s multiple comparisons (*Il23* in e, g, and *Tnf* and *Tjp1* in **h**); two-way ANOVA with Bonferroni’s multiple comparisons (**d**); or unpaired *t* test (**j**).

To validate that eosinophil degranulation-induced small intestinal damage exacerbated psoriatic inflammation, we evaluated the development of psoriatic dermatitis in mice lacking eosinophils. Ideally, we would use mice with selective eosinophil deficiency in the gastrointestinal tract; however, *Ccr3*^-/-^ mice with defective eosinophil recruitment into the small intestine^36^ had increased eosinophil levels in the blood. Thus, IMQ treatment could markedly reduce eosinophils in the blood, which was not observed in WT mice (Supplementary Fig. 7). Therefore, we used ∆dblGATA (∆EO) mice, which were genetically engineered to lack eosinophils (Fig. 5a) and had no significant reductions in other immune cell populations^11^. Additionally, in these mice, the expression of genes linked to inflammation and barrier integrity in the small intestine was similar to that in wild-type mice, except for a significant decrease in *Il17a* and an increase in *Ocln* (Supplementary Fig. 8a). Treatment of ∆EO mice with topical IMQ resulted in psoriatic symptoms (Supplementary Fig. 8b–d). However, IMQ-induced changes in skin thickness and histology were attenuated in ΔEO mice compared with wild-type mice (Fig. 5b–e). IMQ-treated ΔEO mice exhibited changes in their small intestinal microenvironment, although these changes were less pronounced than those in wild-type mice, suggesting that factors other than eosinophils contributed to intestinal inflammation in the IMQ-induced psoriasis model (Supplementary Fig. 8e, f**)**. The mRNA expression of tight junction factors was similar between control and IMQ-treated ΔEO mice (Supplementary Fig. 8f), and serum and stool levels of EPX and serum calprotectin levels were lower in IMQ-treated ΔEO mice than in their wild-type counterparts (Fig. 5f, g). Accordingly, the IMQ-treated ΔEO mice had lower *Tnf* expression and higher *Cldn1*, *Cldn4*, *Ocln*, and *Muc2* expression in the small intestine than IMQ-treated wild-type mice (Fig. 5h).

Intravenous administration of in vitro-generated BMEOs to mice subjected to inflammatory challenge enables the in vivo evaluation of eosinophil-mediated effects^11,37^. We reconstituted eosinophils in ΔEO mice by intravenously injecting BMEOs 2 days before IMQ treatment (Fig. 5a). The adoptive transfer of BMEOs into IMQ-treated ΔEO mice increased eosinophil numbers in the small intestine (Supplementary Fig. 9). BMEO transfer reversed both the attenuation of skin inflammation and the reductions in stool EPX and calprotectin levels caused by eosinophil ablation (Fig. 5b–g). Moreover, BMEO transfer increased *Il1b* expression and decreased the mRNA expression of tight junction molecules in the small intestine. This effect was accompanied by lower protein expression of occludin and mucin2 in the small intestine than in IMQ-treated ΔEO mice (Fig. 5h–j).

### Eosinophil degranulation and inflammatory changes in the small intestine are induced in a TLR7-dependent manner

**Fig. 6:**
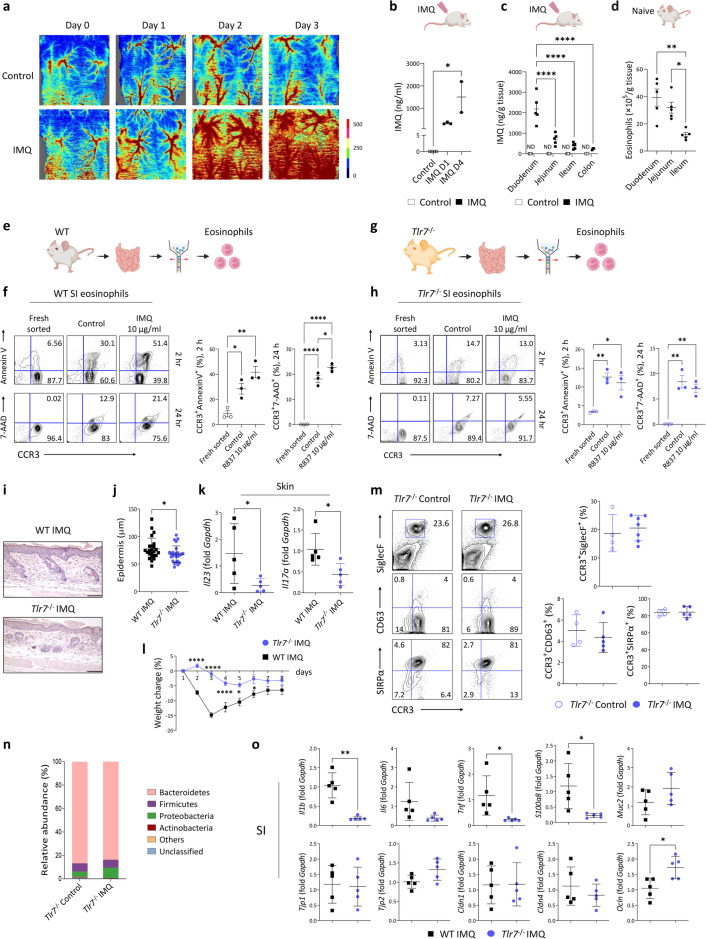
Eosinophil degranulation and inflammatory changes in the small intestine in mice with psoriatic skin inflammation are induced in a TLR7-dependent manner. **a** Representative images of blood perfusion on the dorsal skin of mice. IMQ, imiquimod. The color density indicates the level of microcirculation. IMQ concentrations in the serum (**b**) and intestine (**c**). ND, not detected. **d** Absolute number of eosinophils in naïve mice. **e**, **g** Schematic of eosinophil sorting from the small intestine (SI). WT, wild-type. **f**, **h** Representative flow cytometry plots (left) and percentages (right) of sorted eosinophils stained with Annexin V^+^ or 7-aminoactinomycin D (7-AAD)^+^ after being cultured with IMQ. **i** Hematoxylin and eosin-stained skin. Scale bars, 124.5 μm. **j** Epidermal thickness. Three distinct regions of each section were analyzed. **k** Quantitative PCR analysis of the skin. **l** Weight change (*n* = 4 or 5/group). **m** Representative flow cytometry plots (left) and percentages (right) of SiglecF^+^, CD63^+^, and SIRPα^+^ eosinophils in the SI. **n** Sequencing of the stool microbiota of the SI (*n* = 3). **o** Quantitative PCR analysis of the SI. The data are presented as the mean ± SD. *P < 0.05, **P < 0.01, and ****P < 0.0001 according to the Kruskal–Wallis test with Dunn’s multiple comparisons (**b**); one-way ANOVA with Tukey’s multiple comparisons (**c**, **d**, **f**, and **h**); Mann–Whitney test (**j**, **k**, and **o**); unpaired *t* test (*Il23* in **k** and *Tnf*, *S100a8*, and *Ocln* in **o**); or two-way ANOVA with Bonferroni’s multiple comparisons (**l**).

We hypothesized that a pathogenic interaction occurred between the skin and small intestine through the bloodstream. While the increase in serum levels of inflammatory mediators in IMQ-treated mice could play a role, we aimed to investigate a direct cause-and-effect relationship between the skin and small intestine. Thus, we examined whether interactions mediated by TLR7 contributed to the interplay between the skin and the small intestine in mice with psoriatic dermatitis. As shown in Fig. 6a, IMQ treatment increased blood perfusion in the dorsal skin compared to that in vehicle-treated sites, as indicated by the red areas in the colored pictures. Additionally, LC‒MS/MS showed an increase in serum IMQ concentrations (Fig. 6b). A systemic increase in IMQ increased the concentrations of IMQ in the intestine (Fig. 6c). However, the concentration of IMQ decreased from the proximal to the distal segments of the gastrointestinal tract, corresponding to the distribution pattern of eosinophils in naïve mice (Fig. 6c, d). Furthermore, increases in the expression of miR-16-5p and miR-21-5p, which are microRNAs (miRNAs) associated with human inflammatory diseases that may function as TLR7 ligands^38,39^, were observed in the small intestine of IMQ-treated mice (Supplementary Fig. 10). A positive correlation was noted between intestinal IMQ concentrations and eosinophil distribution in the intestine, and the correlation coefficient was 0.8227 (Supplementary Fig. 11). To test whether small intestinal eosinophils degranulated in a TLR7-dependent manner, we sorted eosinophils from the small intestine (Supplementary Fig. 12) and treated them with IMQ. The expression of the apoptosis marker Annexin V and the death-associated marker 7-AAD was increased in wild-type eosinophils after 2 h and 24 h, respectively (Fig. 6e, f). However, small intestinal eosinophils lacking TLR7 (*Tlr7*^-/-^) did not exhibit signs of apoptosis or cell death after IMQ treatment (Fig. 6g, h), demonstrating that cytolytic death and degranulation of small intestinal eosinophils were dependent on TLR7.

Next, we investigated whether the reduction in small intestinal eosinophils and inflammatory changes induced by psoriatic inflammation were dependent on TLR7. *Tlr7*^−/−^ mice developed IMQ-induced psoriatic dermatitis (Supplementary Fig. 13a–c). However, the severity of the disease was lower than that in wild-type mice (Fig. 6i–l). Notably, *Tlr7*^−/−^ mice did not exhibit changes in the number or expression of CD63 or SIRPα in small intestinal eosinophils following IMQ administration (Fig. 6m). Consistently, no signs of microbial compositional changes were observed between untreated and IMQ-treated *Tlr7*^-/-^ mice (Fig. 6n), despite noticeable differences compared to the bacterial composition observed in the control group of wild-type mice (Fig. 1h). IMQ-treated *Tlr7*^-/-^ mice had lower *Tjp2* and *Cldn4* expression in the small intestine than untreated *Tlr7*^-/-^ mice (Supplementary Fig. 13d), suggesting that factors independent of TLR7 might be involved in intestinal barrier damage. However, IMQ-treated *Tlr7*^-/-^ mice had higher *Ocln* expression and lower inflammatory cytokine levels in the small intestine than IMQ-treated wild-type mice (Fig. 6o). These findings suggest that a TLR7-dependent pathogenic response in the small intestine plays a primary role in promoting psoriatic inflammation.

## Discussion

Patients with psoriasis often exhibit dysbiosis of the gut microbiota and an increased incidence of inflammatory bowel disease^40,41^, indicating a link between the immune responses in the skin and gut. An imbalance in intestinal bacteria contributes to the development or exacerbation of psoriatic inflammation rather than reflecting inflammatory responses in the skin and intestine^42,43^. Our findings suggest that alterations in the intestinal microenvironment require communication between the skin and intestine, which is facilitated by the development of psoriatic dermatitis. IMQ-induced psoriatic dermatitis reproduces the systemic features of psoriasis, including elevated serum levels of inflammatory mediators. Increases in the levels of only 3 of the 40 analyzed inflammatory mediators in the serum were observed following intraperitoneal administration of IMQ (Supplementary Fig. 4e). In contrast, dermal application of IMQ induced robust systemic inflammation, and 11 inflammatory mediators were increased in mice with IMQ-induced psoriatic inflammation (Fig. 1j). Accordingly, over the course of topical IMQ treatment, we observed increases in the serum concentrations of IMQ and detected its presence in the small intestine. These findings suggest that proinflammatory cytokines, which are frequently increased in the serum of patients with progressive psoriasis^44^, can infiltrate the small intestine and disrupt homeostatic immune responses. Markedly higher IMQ levels were observed in the small intestine than in the large intestine. This concentration disparity had discernible effects on the expression of inflammatory mediators and the composition of the microbiome in the small intestine, whereas the large intestine remained relatively unaffected. This variation could be attributed to the extensive microvascular blood flow that supports digestion and nutrient absorption at the absorptive surface of the small intestinal mucosa^9^.

Although TLR7 expression is low in the gut epithelium^45^, the small intestine harbors various TLR7-expressing cells^46^. Additionally, the small intestine has a unique composition of immune cells characterized by abundant levels of resident eosinophils^47,48^. IMQ treatment reduced the viability of small intestinal eosinophils isolated from wild-type mice but not from *Tlr7*^-/-^ mice, suggesting that TLR7-dependent exocytic death occurred in small intestinal eosinophils. Topical IMQ treatment induces IL-23/IL-17 axis-dependent inflammatory responses in the skin in mice^16^. However, we did not examine the expression of genes involved in Th17 cell differentiation in the small intestine after topical IMQ treatment. Thus, TLR7 ligation in small intestinal eosinophils may not play a role in the direct activation of the IL-23/IL-17 immune response. Previous studies have shown that eosinophil TLR7 ligation accelerates the clearance of respiratory syncytial virus and the generation of superoxide^49,50^. Therefore, we propose that TLR7 activation in eosinophils damages the small intestine, leading to systemic inflammatory responses. In addition to IMQ, noncoding miRNAs, which are increased within the gastrointestinal tract of mice with psoriatic inflammation, can function as endogenous TLR7 ligands. Consistent with this concept, the levels of miRNAs that originate from both hematopoietic and nonhematopoietic intestinal cells were increased in response to inflammatory changes^51^. Although a decreasing IMQ gradient from the proximal to the distal regions of the intestine suggested a distribution similar to that of skin-derived inflammatory mediators, the presence of IMQ in the small intestine suggests that small intestinal eosinophils may be activated by IMQ. Distinguishing the effects of IMQ on eosinophil activation from those caused by increased inflammatory cytokine levels in the small intestine is challenging. Therefore, the use of TLR7-ligand-independent psoriasis models that induce systemic inflammatory responses may be effective in directly determining the role of TLR7-dependent eosinophil activation in the small intestine.

Although we did not perform an in-depth analysis, small intestinal pDCs express high levels of TLR7^52^ and exhibit a decrease similar to that in eosinophils after topical IMQ application. Notably, pDCs perform two opposing functions by activating autoreactive T cells in response to the presence of nucleic acids and inducing the differentiation of Treg cells^53^. Therefore, the psoriasis-induced reduction in small intestinal pDCs might have an impact on the small intestinal immune environment. Further investigations are necessary to confirm the presence of endogenous TLR7 ligands in the small intestine of patients with psoriasis. Additionally, these studies should aim to clarify how pDCs and other cells expressing TLR7 in the small intestine respond to ligands triggered by psoriatic inflammation.

In inflammatory conditions, such as parasitic helminth infection, allergic disease, and tissue injury, eosinophils act as cytotoxic effector cells by releasing granule-derived cytotoxic proteins^54^. However, under steady-state conditions, eosinophils are particularly abundant in the small intestinal lamina propria and maintain homeostatic responses without apparent degranulation^34,55^. Eosinophils are more abundant than any other immune cells in the small intestines of WT mice and contain approximately 300, 50, and 20 times more eosinophils than the skin, large intestine, and blood, respectively (Supplementary Fig. 7). Accordingly, if psoriatic dermatitis exerts systemic effects that result in eosinophil degranulation and consequent tissue damage, it is reasonable to expect the damage to occur first, or even primarily, in the small intestine. Eosinophils express a diverse array of receptors that enable them to respond to external stimuli. Furthermore, they contain lipid mediators, cytotoxic proteins, and cytokines that collectively exert effects in a pathological context^48,55^. These unique features of eosinophils could accelerate psoriatic dermatitis, which was strongly supported by our in vivo study in which mice were predisposed to eosinophil degranulation. Small intestinal eosinophils express higher levels of SIRPα, a cognate receptor for CD47 that is expressed in epithelial cells, than blood and bone marrow eosinophils, and the SIRPα/CD47 interaction promotes eosinophil survival by inhibiting degranulation^34^. Compared with *Epx*^+/+^*Sirpa*^fl/fl^ mice, *Epx*^Cre/+^*Sirpa*^fl/fl^ mice exhibited accelerated small intestinal eosinophil degranulation, increased small intestinal damage, and exacerbated psoriatic dermatitis. Since the degranulation of skin eosinophils accelerates psoriasis pathogenesis^30^, it is necessary to address the role of small intestinal eosinophils in the exacerbation of psoriatic inflammation compared to those in the skin. Although our study demonstrated an association between psoriatic skin inflammation and small intestinal eosinophil activation, we were unable to experimentally establish a direct link between these two phenomena. Mice that were selectively deficient in small intestinal eosinophils while maintaining eosinophil levels in other tissues comparable to those of wild-type mice were not available for our study. Thus, we anticipated that psoriatic dermatitis would be alleviated in these mice compared with their wild-type counterparts.

Although the small intestine is rich in substances that can stimulate eosinophils^56^, endogenous activating signals are insufficient to induce eosinophil degranulation under steady-state conditions^47^. Therefore, our findings suggest that external stimuli entering systemic circulation are required to induce inflammatory changes in the intestine, which can further promote the inflammatory responses observed during psoriasis. Intraperitoneal IMQ injection in our mice did not change the number of eosinophils or the expression of cytokines or tight junction molecules in the small intestine. Therefore, we propose that the development of psoriatic dermatitis, which is accompanied by systemic inflammation, could be an external stimulus that induces environmental changes in the small intestine. Consistent with this concept, intestinal helminth-specific Th2 cells infiltrate the skin of infected mice by migrating through the blood^57^, indicating an interconnected immune response in the skin and gut. We previously observed increased eosinophil degranulation in human psoriatic skin lesions^30^. Although we did not observe changes in the small intestine in patients with psoriasis, changes associated with eosinophil activation may contribute to the pathogenesis of psoriasis. Therefore, reinforcing the homeostatic role of small intestinal eosinophils may be beneficial for treating psoriasis.

### Supplementary information

Supplementary information

## Data Availability

The datasets generated by this study are available from the corresponding author upon request. The RNA sequencing data have been deposited in the Gene Expression Omnibus repository under accession code GSE237610.
