# Supplementary material for: TLR7-dependent eosinophil degranulation links psoriatic skin inflammation to small intestinal inflammatory changes in mice
Source: Exp Mol Med. 2024 May 1;56(5):1164–77. doi: 10.1038/s12276-024-01225-y (PMC11148187; doi:10.1038/s12276-024-01225-y)
Supplement: Supplementary file 1 — Supplementary information [file 12276_2024_1225_MOESM1_ESM.pdf]

Supplementary Materials and Methods

Intraperitoneal injection of imiquimod (IMQ)

IMQ was systemically administered as previously described[1]. Briefly, R837 (InvivoGen, San Diego, CA, USA) was dissolved in PBS at a concentration of 1 mg/ml and injected intraperitoneally (10 mg/kg) daily for a week.

Quantitative PCR analysis of microRNAs (miRNAs)

miRNeasy kit (Qiagen, Hilden, Germany) was used to extract and purify miRNA from the small intestine. cDNA of miRNA was synthesized using miRCURY LNA RT kit (Qiagen). miRCURY LNA miRNA PCR assay primers were used to measure miR-16-5p and miR-21-5p (Qiagen; cat# YP00205702 and YP00204230, respectively). The housekeeping primers for miRNA experiments were U6 small nuclear RNA (Qiagen; cat# YP02119464).

References

1. Kiyohara, H. et al. Toll-Like Receptor 7 Agonist-Induced Dermatitis Causes Severe Dextran Sulfate Sodium Colitis by Altering the Gut Microbiome and Immune Cells. *Cell Mol Gastroenterol Hepatol* **7**, 135-156 (2019).

Supplementary Figures and Figure legends

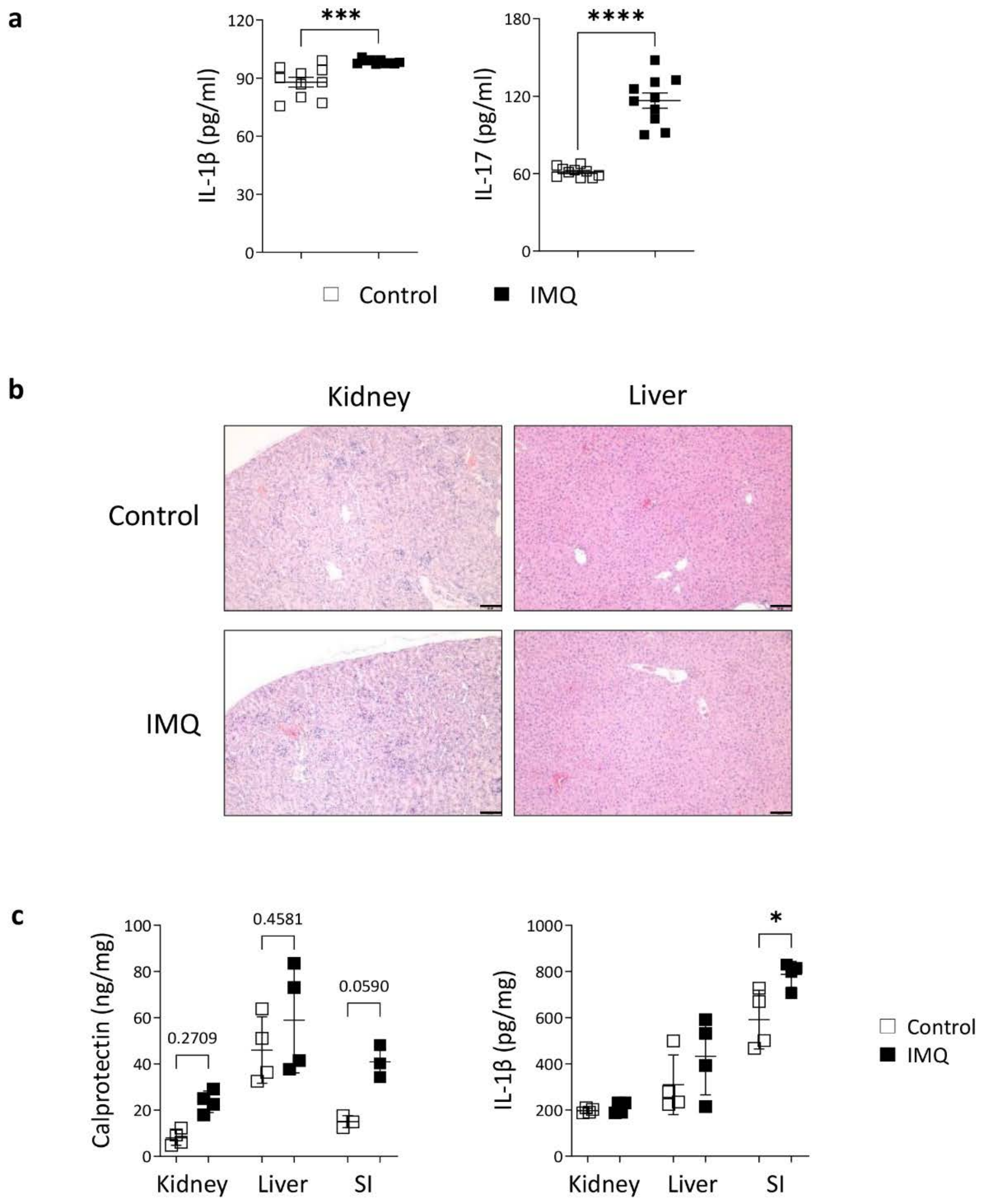

Supplementary Fig. 1. Inflammatory responses of imiquimod-treated mice. (a) Serum levels of IL-1β and IL-17. Quantikine mouse IL-1β and IL-17 ELISA kits (R&D Systems, Minneapolis, MN, USA; cat# MLB00C-1 and M1700-1, respectively) were

used to according to the manufacturer’s instructions. (b) Hematoxylin and eosin-stained kidney and liver. Scale bars, 100 μm. (c) Calprotectin and IL-1β levels in the kidney, liver, and small intestine (SI). Data are presented as the mean ± SD. *$P < 0.05$, ***$P < 0.001$, and ****$P < 0.0001$ by unpaired *t*-test (a) or one-way ANOVA with Bonferroni's multiple comparisons (c).

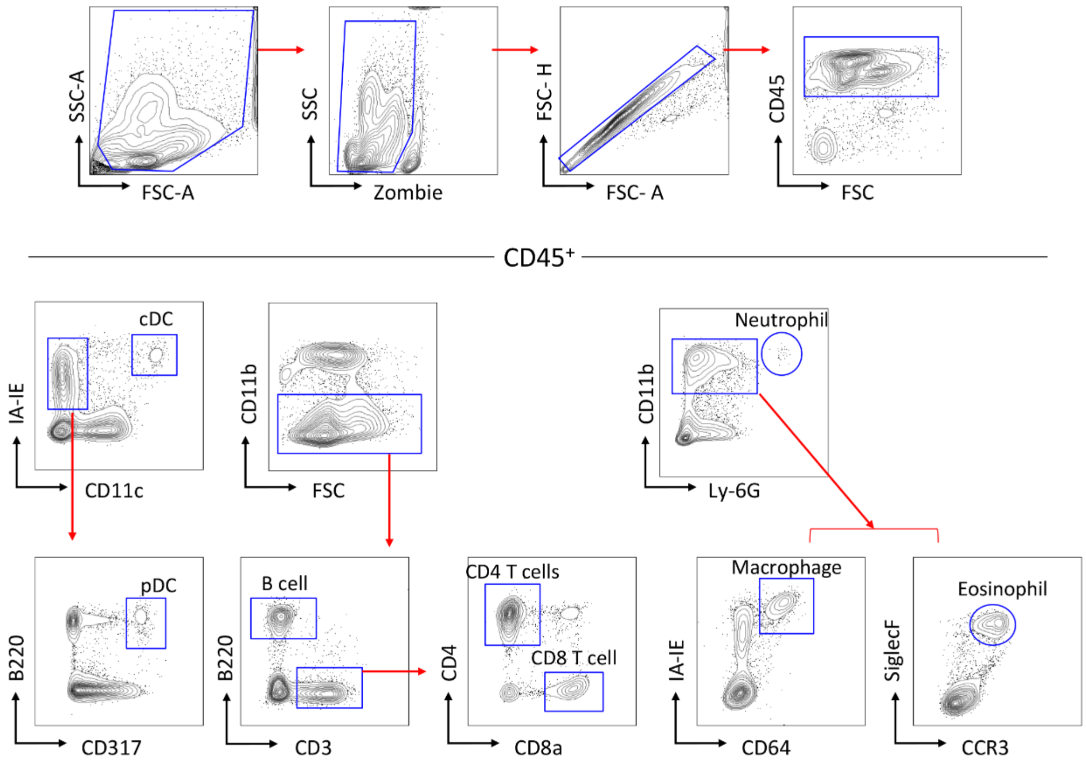

Supplementary Fig. 2. Representative flow cytometry plots depicting the gating strategy for small intestinal immune cells.

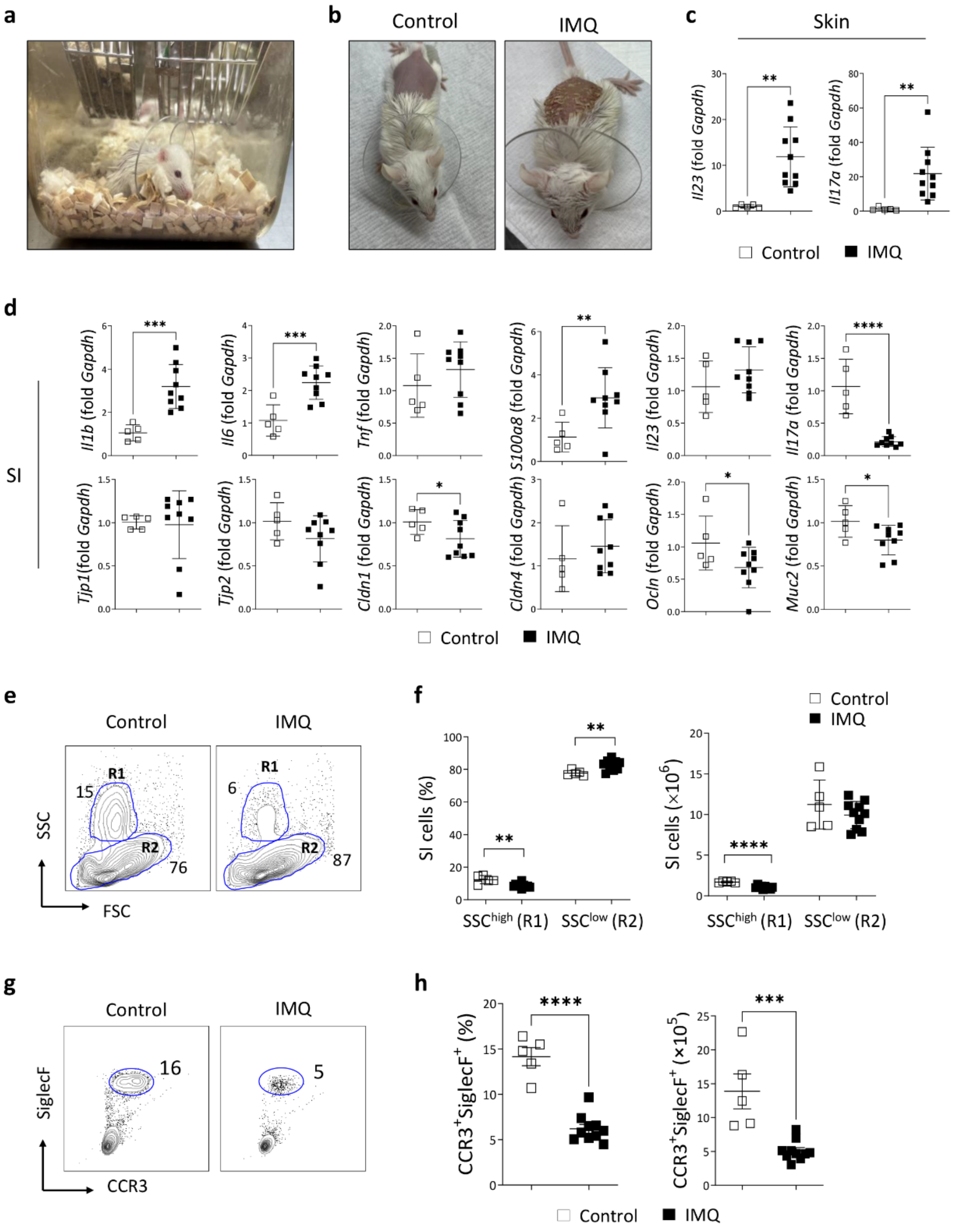

Supplementary Fig. 3. Pathologic changes induced in imiquimod (IMQ)-treated mice wearing Elizabethan collars. (a) A photograph of mouse housed while wearing an Elizabethan collar. (b) Photographs of mouse skin treated with vehicle cream (control) or IMQ while wearing an Elizabethan collar. (c) Quantitative PCR analysis in the skin. (d) Quantitative PCR analysis in the small intestine (SI). (e) The forward

scatter (FSC) and side scatter (SSC) populations of cells in the SI. SSC^{high} and SSC^{low} cells are indicated as R1 and R2, respectively. (f) Percentage (left) and absolute number (right) of SSC^{high} and SSC^{low} cells in the SI. (g) Representative flow cytometry plots of eosinophils in the SI. (h) Percentage (left) and absolute number (right) of eosinophils in the SI. Data are presented as the mean ± SD. $*P < 0.05$, $**P < 0.01$, $***P < 0.001$, and $****P < 0.0001$ by unpaired *t*-test or Mann–Whitney test (absolute number of eosinophils in h).

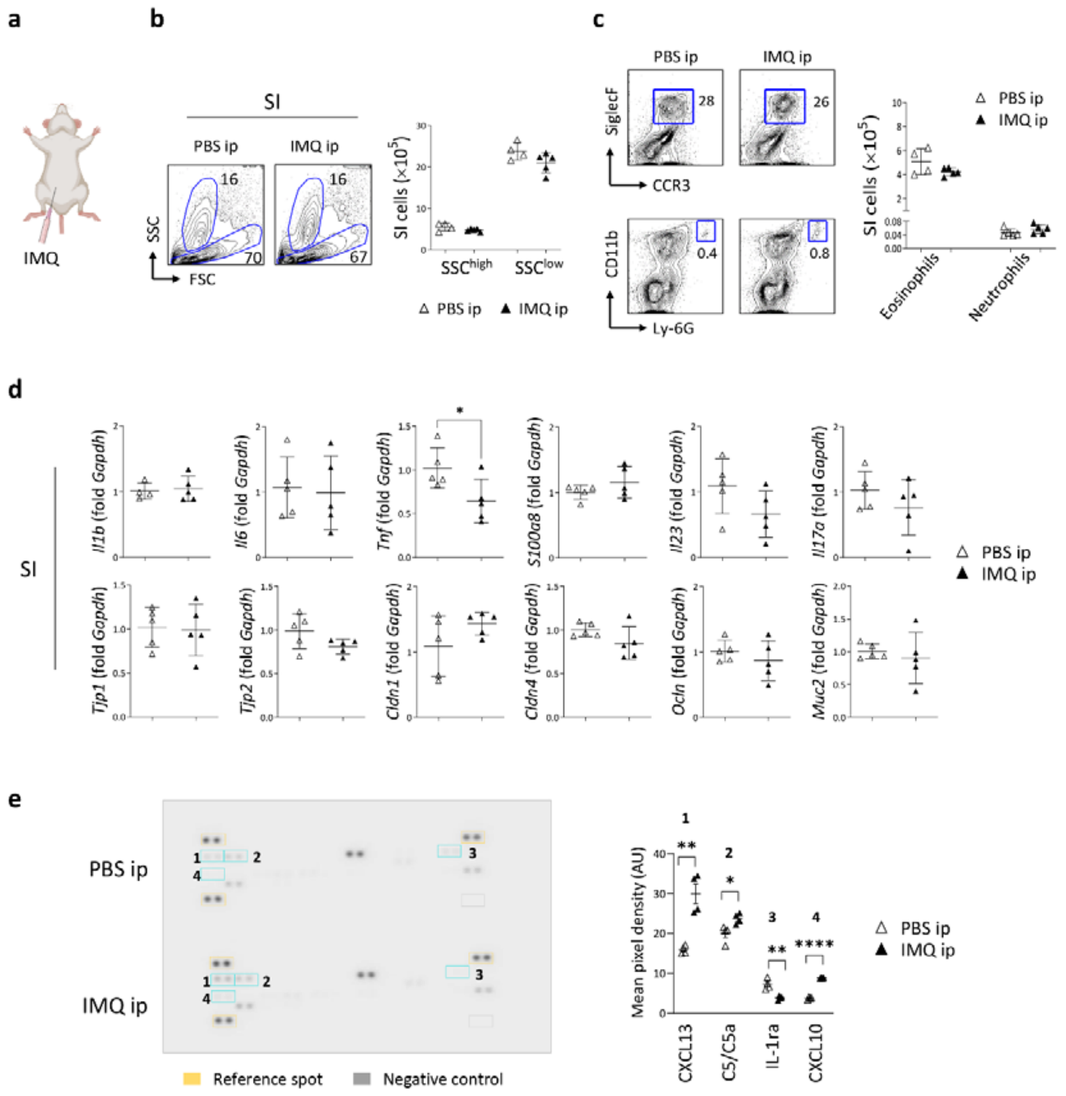

Supplementary Fig. 4. Cell frequency and small intestinal mRNA expression in mice intraperitoneally injected with imiquimod (IMQ). (a) Schematic of intraperitoneal (ip) administration of IMQ. (b) Representative flow cytometry plots (left) and absolute numbers (right) of SSC^{high} and SSC^{low} cells in the small intestine (SI). (c) Representative flow cytometry plots (left) and absolute numbers (right) of eosinophils and neutrophils in the SI. (d) Quantitative PCR analysis in the SI. (e) Picture of cytokine array membrane (left) and the quantified density of indicated spots (right) in the serum. Data are presented as the mean ± SD. **P* < 0.05, ***P* < 0.01, and *****P* < 0.0001 by unpaired *t*-test.

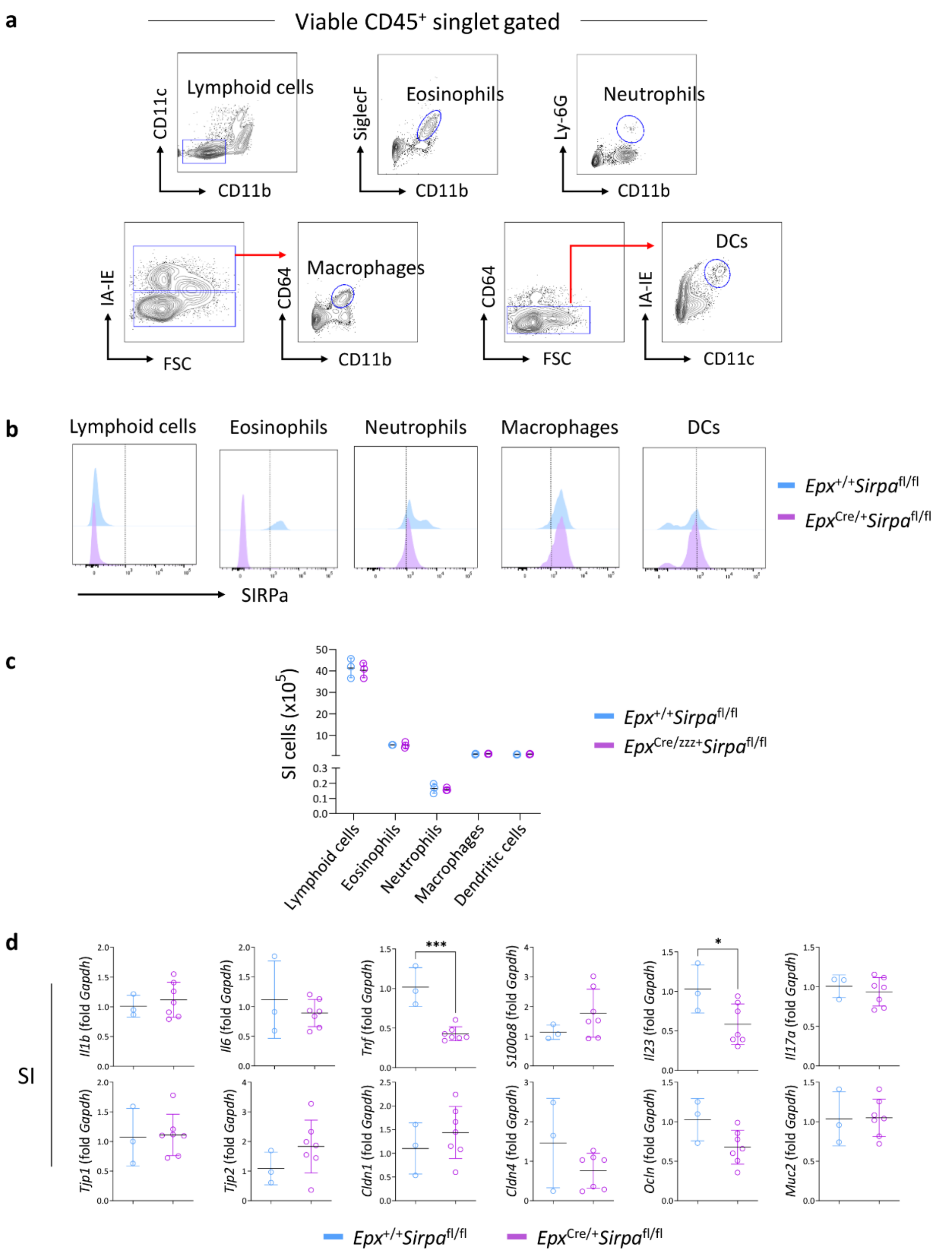

Supplementary Fig. 5. Deletion of signal regulatory protein alpha (SIRPα) in eosinophils. (a) Representative flow cytometry plots depicting the gating strategy for lymphoid cells, eosinophils, neutrophils, macrophages, and dendritic cells (DCs) in the small intestine (SI). (b) Surface expression of SIRPα on small-intestinal lymphoid cells,

eosinophils, neutrophils, macrophages, and DCs in *Epx*$^{+/+}$*Sirpa*$^{fl/fl}$ and *Epx*$^{Cre/+}$*Sirpa*$^{fl/fl}$ mice. (c) Absolute numbers of immune cells in the SI. (d) Quantitative PCR analysis in the SI. Data are presented as the mean ± SD. *$P < 0.05$ and ***$P < 0.001$ by unpaired *t*-test.

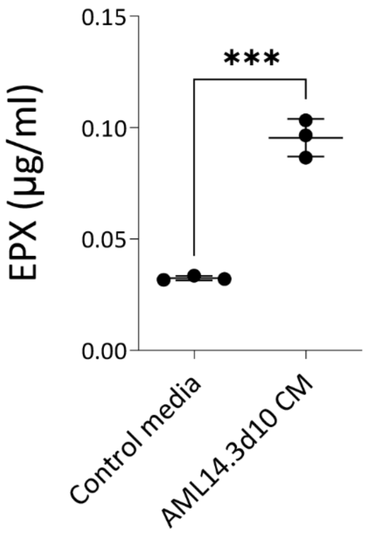

Supplementary Fig. 6. Eosinophil peroxidase (EPX) concentration in culture supernatants. Data are presented as the mean ± SD. ****P* < 0.001 by unpaired *t*-test.

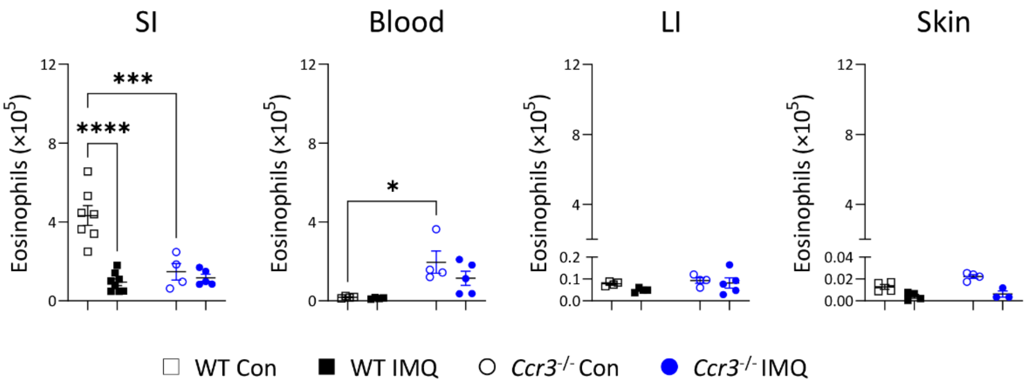

Supplementary Fig. 7. Eosinophils in the small intestine (SI), blood, large intestine (LI), and skin of imiquimod (IMQ)-treated wild-type (WT) and *Ccr3*$^{-/-}$ mice. Data are presented as the mean ± SD. *$P < 0.05$, ***$P < 0.001$, and ****$P < 0.0001$ by one-way ANOVA with Tukey's multiple comparisons.

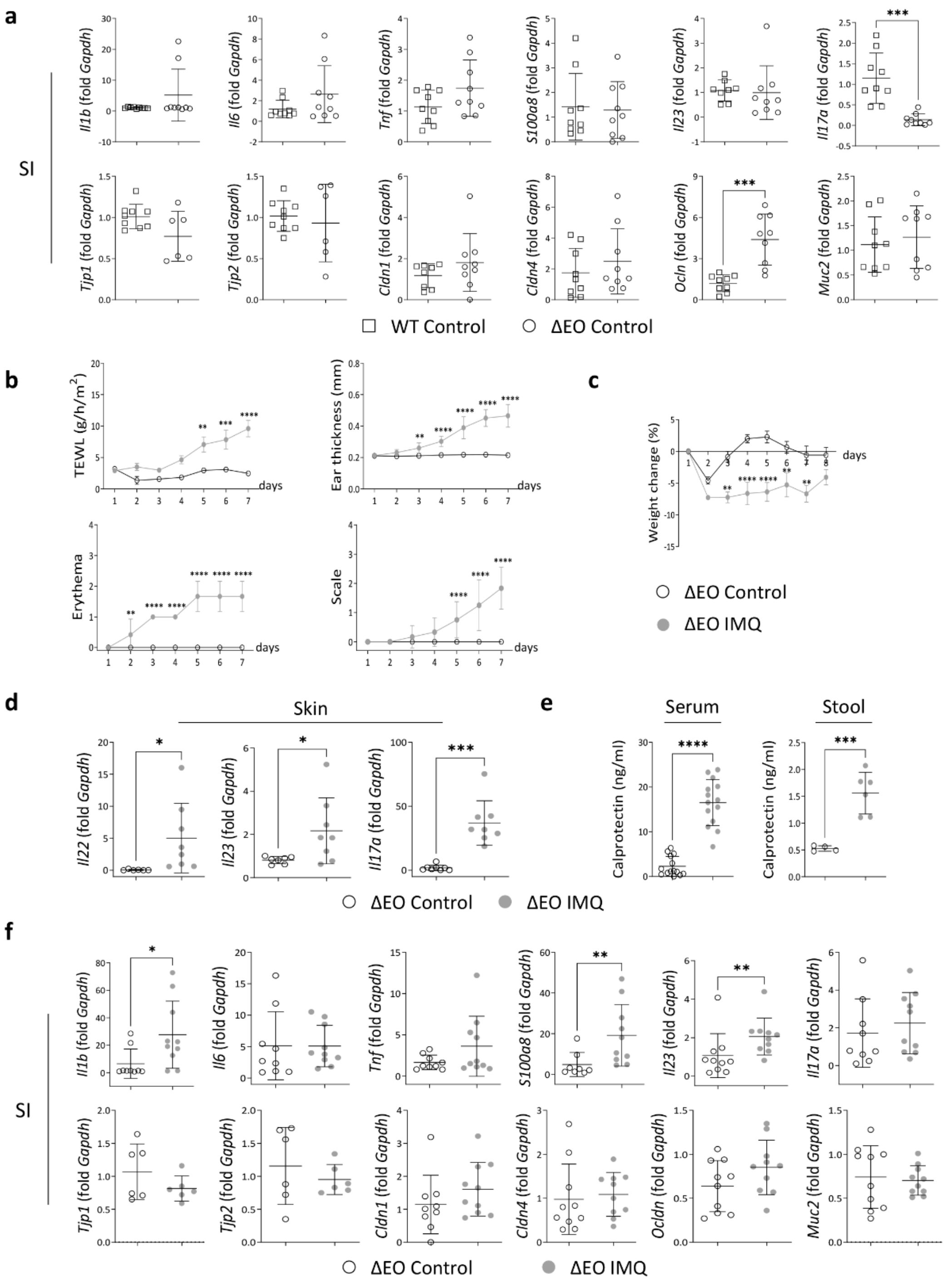

Supplementary Fig. 8. Pathologic changes induced in imiquimod (IMQ)-treated ΔdblGATA (ΔEO) mice. (a) Quantitative PCR analysis in the small intestine (SI). (b) Transepidermal water loss (TEWL), ear thickness, erythema, and scaling (n = 8–12 per group). (c) Weight change (n = 4 per group). (d) Quantitative PCR analysis in the skin. (e) Concentrations of calprotectin in serum (left) and stool (right). (f) Quantitative PCR

analysis in the SI. Data are presented as the mean ± SD. *$P < 0.05$, **$P < 0.01$, ***$P < 0.001$, and ****$P < 0.0001$ by unpaired *t*-test (a, d, and e), two-way ANOVA with Bonferroni's multiple comparisons (b and c), or Mann–Whitney test (*Il17a* in d, serum calprotectin in e, and f).

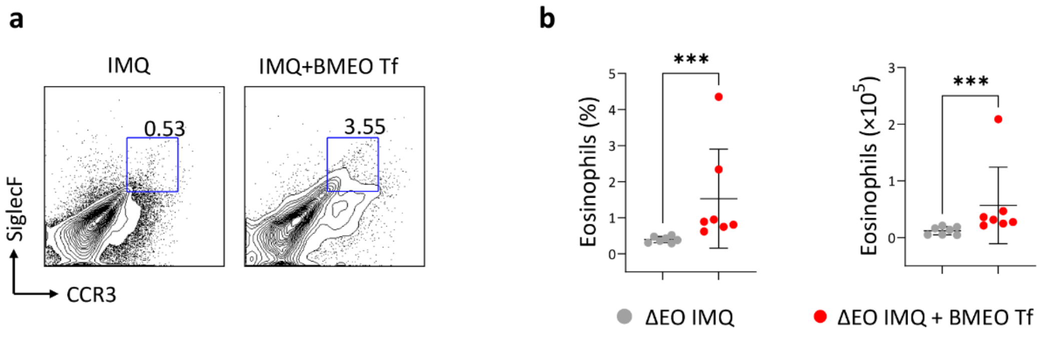

Supplementary Fig. 9. Eosinophils in the small intestines of imiquimod (IMQ)-treated ΔdblGATA (ΔEO) mice with adoptive transfer of bone marrow-derived eosinophils (BMEOs). (a) Representative flow cytometry plots of eosinophils in the small intestine. $CD45^+$ singlet cells were gated. (b) Percentages (left) and absolute numbers (right) of eosinophils in the small intestine. Data are presented as the means ± SD. ****P* < 0.001 by Mann–Whitney test.

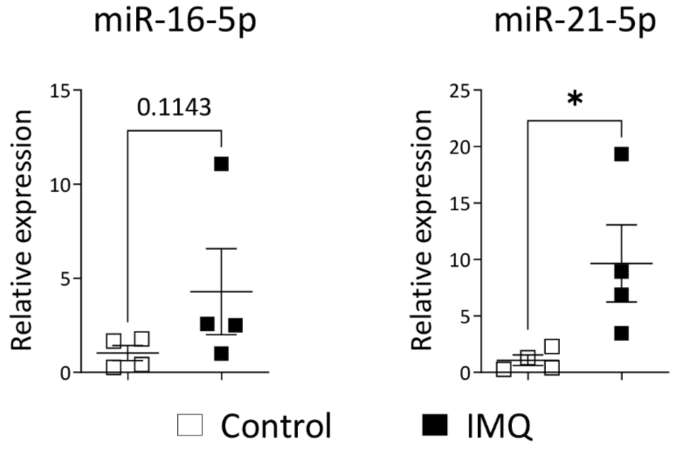

Supplementary Fig. 10. Small intestinal microRNA (miRNA) changes induced in imiquimod (IMQ)-treated wild-type mice. Quantitative PCR analysis of miRNA changes in the small intestine. Data are presented as the mean ± SD. **P* < 0.05 by Mann–Whitney test.

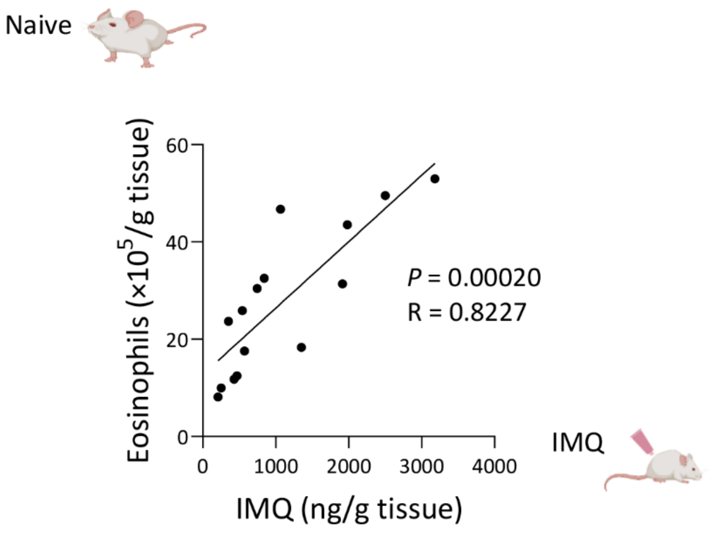

Supplementary Fig. 11. Distribution of eosinophils in wild-type mice. Correlation between small intestinal eosinophil counts in naïve mice and small intestinal imiquimod (IMQ) levels following topical IMQ treatment. Data are presented as the mean ± SD.

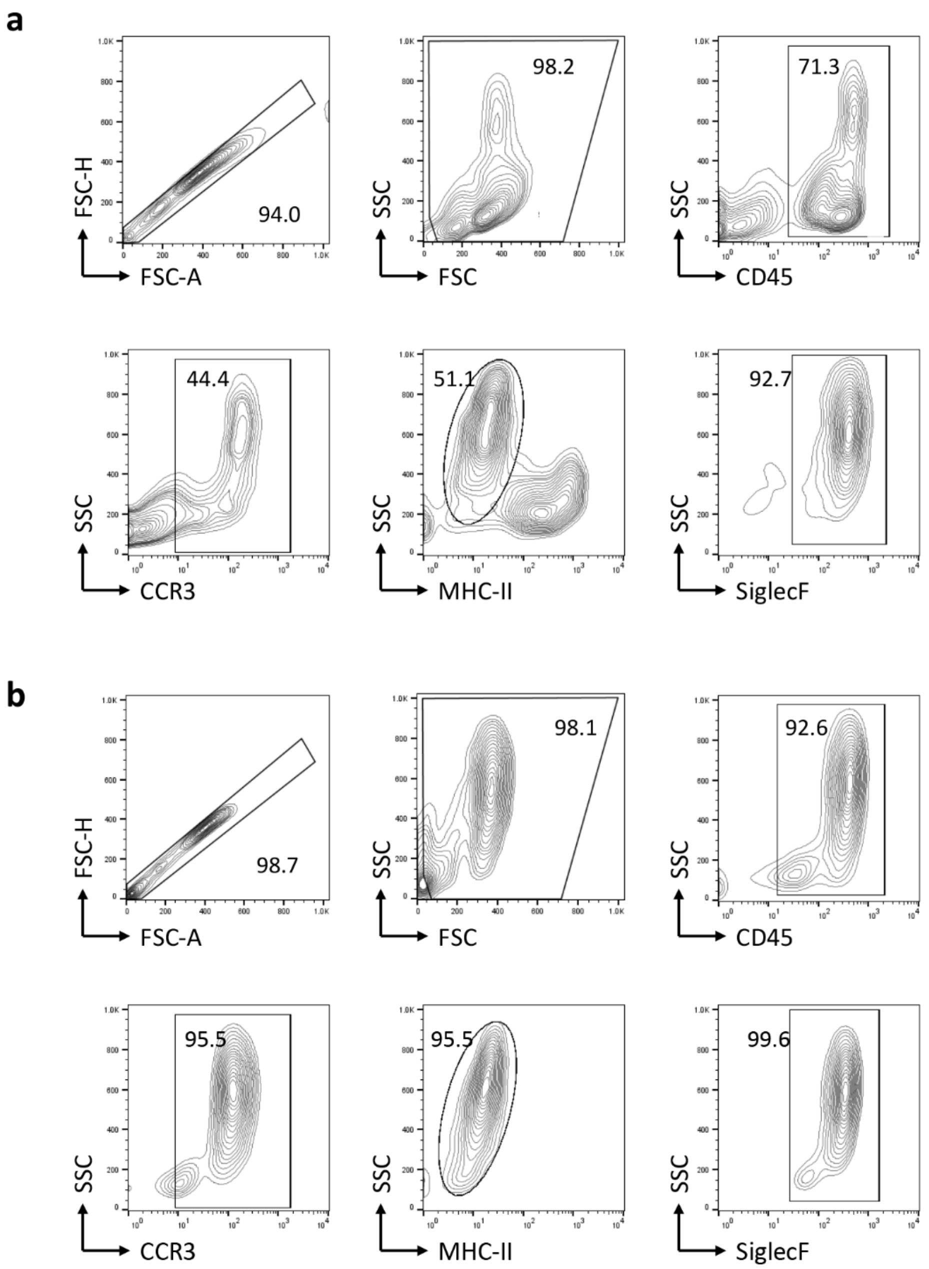

Supplementary Fig. 12. Sorting strategy for small intestinal eosinophils. (a) Representative flow cytometry plots depicting the gating strategy for eosinophil sorting from the small intestine. (b) Representative flow cytometry plots depicting small intestinal eosinophil purity after sorting.

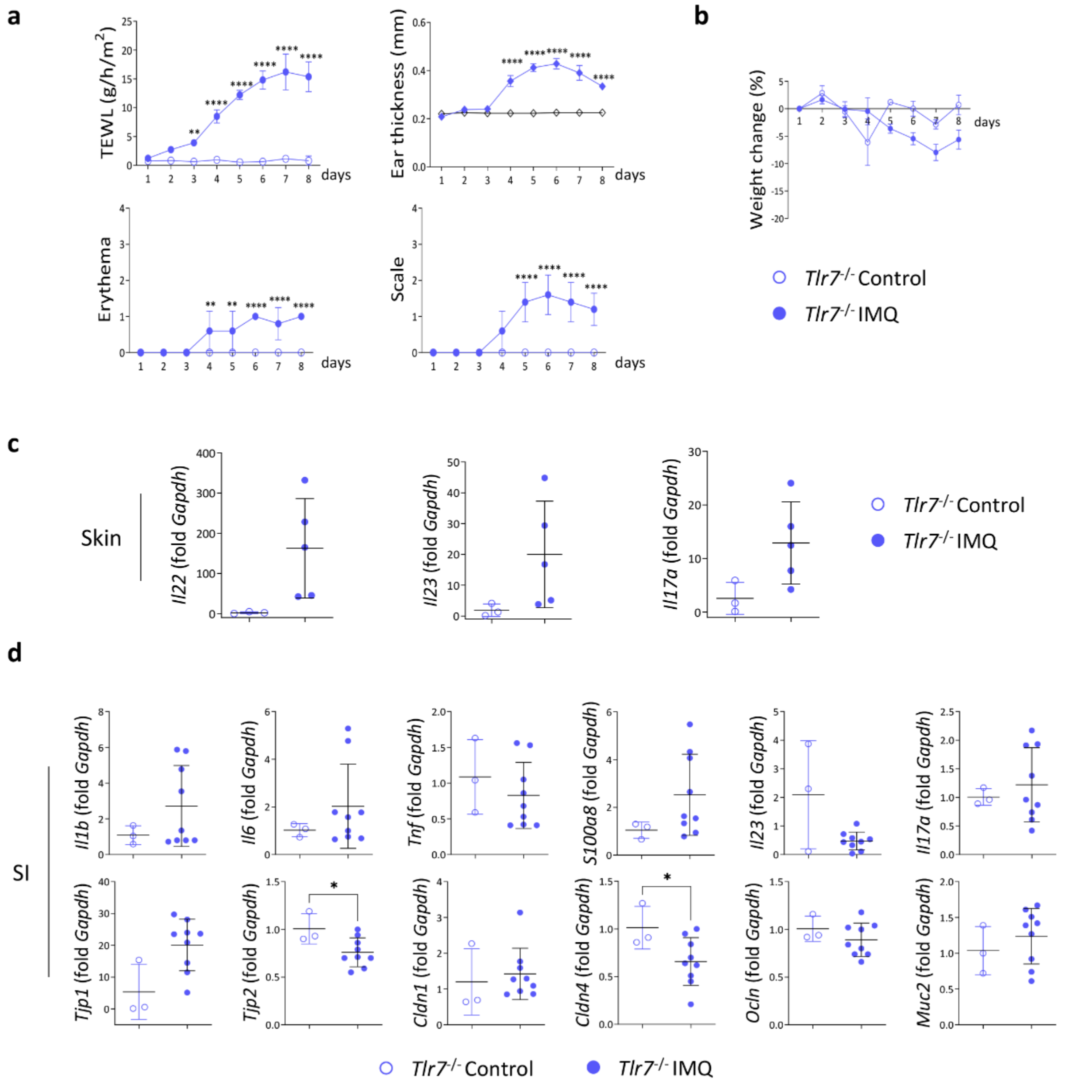

Supplementary Fig. 13. Pathologic changes induced in imiquimod (IMQ)-treated *Tlr7*$^{-/-}$ mice. (a) Transepidermal water loss (TEWL), ear thickness, erythema, and scaling (n = 4–5 per group). (b) Weight change (n = 4 per group). (c) Quantitative PCR analysis in the skin. (d) Quantitative PCR analysis in the small intestine (SI). Data are presented as the mean ± SD. **P* < 0.05, ***P* < 0.01, and *****P* < 0.0001 by two-way ANOVA with Bonferroni's multiple comparisons (a) or unpaired *t*-test (d).

Supplementary Table

Supplementary Table 1. Primer sequences for real-time PCR

Target gene	Primer sequence
Il1b	Forward: 5′- GCA ACT GTT CCT GAA CTC AAC T -3′<br>Reverse: 5′- ATC TTT TGG GGT CCG TCA AC -3′
Il6	Forward: 5′- TAG TCC TTC CTA CCC CAA TT -3′<br>Reverse: 5′- TTG GTC CTT AGC CAC TCC TTC -3′
Tnf	Forward: 5′- CCT GTA GCC CAC GTC GTA G -3′<br>Reverse: 5′- GGG AGT AGA CAA GGT ACA ACC C -3′
S100a8	Forward: 5′- TTC CTT GCG ATG GTG ATA -3′<br>Reverse: 5′- ATG ACT TTA TTC TGT AGA CA -3′
Il22	Forward: 5′- ACC AGA ACA TCC AGA AT -3′<br>Reverse: 5′- CTC AGA CGC AAG CAT TTC -3′
Il23	Forward: 5′- CTA AGA GAA GAA GAG GAT GAA GAG -3′<br>Reverse: 5′- CTG GCT GTT GTC CTT GAG -3′
Il17a	Forward: 5′- GAC TTC CTC CAG AAT GTG AA -3′<br>Reverse: 5′- TGG AAC GGT TGA GGT AGT -3′
Tjp1	Forward: 5′- ACC TCT ACT CTA CGA CAT -3′<br>Reverse: 5′- GTG GAA CTT GCT CAT AAC -3′
Tjp2	Forward: 5′- GTG GAG TGG TTC GGT TGA -3′<br>Reverse: 5′- TGA GTG TAG TTG AGC AGG TC -3′
Cldn1	Forward: 5′- TCT GCC ACT TCT CAC TTC -3′<br>Reverse: 5′- GCC TAT ACC CTT GCT CTC -3′
Cldn4	Forward: 5′- TGG ATG GAC GGG TTT GAG -3′<br>Reverse: 5′- CAC ACT GGG CTG CTT CTA -3′
Ocln	Forward: 5′- TTG GCT ACG GAG GTG GCT ATG -3′<br>Reverse: 5′- TTT GGC TGC TCT TGG GTC TGT -3′
Muc2	Forward: 5′- CTC TGC TGT CTC CGT CAT -3′<br>Reverse: 5′- CAC TGG TCT TCT CCT CCT T -3′
Ccr3	Forward: 5′- CAG TGC TTT TGG GTG TTT GTC -3′<br>Reverse: 5′- GAT TTC TAG GGT CTG TGT GCC -3′
Prg2	Forward: 5′- TGA AAC TTC TGA CTC CAA AAG CC -3′<br>Reverse: 5′- CGG CAT TAG CTC TTC CCC T -3′
Gapdh	Forward: 5′- CTG GTA TGA CAA TGA ATA CGG -3′<br>Reverse: 5′- GCA GCG AAC TTT ATT GAT GG -3′
TNF	Forward: 5′- CCT GTG AGG AGG ACG AAC AT -3′<br>Reverse: 5′- GGT TGA GGG TGT CTG AAG GA -3′
TJP1	Forward: 5′- AAT GAA TGA TGG TTG GTA TGG T -3′<br>Reverse: 5′- GAC AGG TAG GAC AGA CGA -3′
TJP2	Forward: 5′- GGA AAG ATG GAA GGG ATG GAT GA -3′<br>Reverse: 5′- ACT GAT GAC GCG GCT GTC -3′

GAPDH	Forward: 5′- CTG GGC TAC ACT GAG CAC C -3′<br>Reverse: 5′- AAG TGG TCG TTG AGG GCA ATG -3′
